# Albumin Supplementation Preserves Systemic Antioxidant Defenses in Critically Ill, Septic Patients: A Prospective Comparative Study

**DOI:** 10.3390/biomedicines14102156

**Published:** 2026-09-24

**Authors:** Kamilla Domokos, Balázs Szirmay, Nelli Farkas, Rita Jakabfi-Csepregi, Lívia Szélig, Csaba Csontos, Krisztina Szalma, István Wittmann, Balázs Sütő

**Affiliations:** 1 Department of Anaesthesia and Intensive Therapy, Medical School, University of Pécs, 7624 Pécs, Hungary; domokos.kamilla@pte.hu (K.D.);; 2 Department of Laboratory Medicine, Medical School, University of Pécs, 7624 Pécs, Hungary; 3 Institute of Bioanalysis, Medical School, University of Pécs, 7621 Pécs, Hungary; 4 2nd Department of Medicine and Nephrology-Diabetes Centre, Medical School, University of Pécs, 7624 Pécs, Hungary

**Keywords:** hypoalbuminemia, albumin supplementation, sepsis, inflammation, redox balance, oxidative stress, antioxidant capacity, reactive oxygen species, critical illness, intensive care

## Abstract

**Background/Objectives**: Sepsis is a life-threatening syndrome characterized by a dysregulated host response to infection, commonly accompanied by hypoalbuminemia (<30 g/L serum albumin level), associated with excessive production of reactive oxygen and nitrogen species, leading to oxidative stress, endothelial dysfunction, and organ injury. Low serum albumin reflects increased vascular permeability, reduced hepatic synthesis, and enhanced proteolysis. It also indicates the loss of albumin’s antioxidant and anti-inflammatory functions beyond oncotic regulation. Hypoalbuminemia may exacerbate this by reducing plasma antioxidant capacity and free radical scavenging, supporting interest in albumin supplementation as a potential therapeutic strategy. **Methods**: This prospective, randomized study investigated the effects of 3-day albumin administration in ICU patients (*n* = 38) with sepsis and hypoalbuminemia by assessing changes in oxidative stress and inflammatory markers. **Results**: Albumin supplementation increased serum albumin (*p* < 0.001) and was associated with higher total antioxidant capacity (Day 3 *p* = 0.045; Day 5 *p* = 0.004), glutathione levels (Day 3 *p* = 0.003; Day 5 *p* = 0.008), and protein sulfhydryl groups (Day 3–5 *p* = 0.004). Albumin-treated patients showed increased urinary concentration of the oxidative stress–related tyrosine isomers ortho- and meta-tyrosine (*p* = 0.004 and *p* = 0.001, respectively). Catalase activity and myeloperoxidase, procalcitonin, and IL-1β levels did not differ between groups, while IL-6 declined over time (*p* = 0.029). **Conclusions**: Based on our results, in septic patients with hypoalbuminemia, short-term albumin supplementation seems to restore serum albumin and improve systemic redox status, as evidenced by increased total antioxidant capacity, glutathione, and protein sulfhydryl levels. However, albumin supplementation did not significantly affect systemic inflammatory markers or cytokine dynamics. Albumin appears to act primarily by modulating oxidative stress, supporting its role as an adjunctive therapy to restore redox homeostasis in sepsis.

## 1. Introduction

Albumin is the most abundant plasma protein, exhibiting a unique set of roles, modulating acid-base balance, maintaining colloid oncotic pressure and endothelial integrity. Beyond its oncotic properties, albumin exerts several biologically relevant functions that may be particularly important in sepsis. These include antioxidant activity mediated by its free thiol group, binding and transport of endogenous and exogenous substances, detoxification of inflammatory mediators, anticoagulant effects and stabilization of the endothelial glycocalyx. Through these mechanisms, albumin may modulate inflammatory responses, oxidative stress and vascular integrity; thus, it has an important role in maintaining physiological reactions in humans [1,2]. Albumin is also a multifunctional plasma protein that transports hormones, fatty acids, peptides, and certain neuropeptides. In sepsis, albumin and neuropeptides may have distinct but complementary roles: albumin supports hemodynamic stability and exerts immunomodulatory effects, whereas neuropeptides may provide protective anti-inflammatory effects against sepsis-related organ dysfunction [3,4]. Albumin supplementation may contribute to the maintenance of organ function in critically ill patients with hypoalbuminemia; however, evidence for its impact on clinical organ-function outcomes remains inconclusive. Hypoalbuminemia may influence the bioavailability and signaling pathways of neuropeptides, which play complex and predominantly anti-inflammatory roles in the modulation of the inflammatory response, thereby representing a potentially promising therapeutic target [5,6].

The antioxidant effect of albumin originates from the structure of the molecule; the reduced Cys34 residue constitutes the largest pool of thiol groups, binding reactive oxygen species (ROS) like hydroxyl radicals, and reactive nitrogen species (RNS) such as nitric oxide and peroxynitrite. In addition, the ligand-binding capacities of albumin also contribute to its antioxidant properties—such ligands are copper (II), iron (II) and bilirubin [7].

Multiple organ dysfunction syndrome is a major cause of mortality in sepsis [8]. An excessive amount of reactive oxygen species is produced through the activation of neutrophils and macrophages, the NADPH oxidase system and mitochondrial damage [9]. At the same time, nitric oxide production increases, forming reactive compounds such as peroxynitrite that damage lipids, proteins, and DNA [9]. An unbalanced redox homeostasis is seen, a shift toward a pro-oxidant state and the depletion of systemic antioxidant defenses [10,11,12].

Despite decades of clinical use and extensive investigation, the optimal indications and timing of albumin supplementation in septic patients remain uncertain [13]. Hypoalbuminemia is common in sepsis and is associated with greater disease severity and worse clinical outcomes [14]. Its development is multifactorial, resulting from endothelial dysfunction and capillary leakage, impaired hepatic synthesis, increased protein catabolism, and dilutional effects caused by large-volume fluid resuscitation [15]. Owing to its diverse molecular characteristics, human albumin supplementation has gained significant attention as a therapeutic choice in critically ill patients [16].

The use of albumin in the management of septic patients has been investigated in several clinical trials, primarily in the context of fluid resuscitation [17,18,19]. Large randomized controlled studies have evaluated albumin administration compared with crystalloid solutions, focusing mainly on mortality outcomes [20]. While some studies suggested potential benefits in specific patient subgroups, overall results have remained inconclusive, and current clinical guidelines recommend albumin mainly as an adjunct in patients requiring substantial amounts of crystalloids [13].

Importantly, most previous clinical investigations have focused on mortality as the primary outcome, whereas the potential biological effects of albumin on inflammatory responses, oxidative stress, and hemodynamic support have received less attention. As a result, the mechanistic clinical impact of albumin administration in septic patients remains incompletely understood.

Therefore, the aim of the present study was to investigate the effects of albumin administration in patients with sepsis who were admitted to the intensive care unit, with particular focus on inflammatory markers and oxidative stress parameters.

## 2. Materials and Methods

### 2.1. Study Objectives and Hypothesis

Although prior studies using mortality as a primary endpoint have reported negative results for albumin administration, these outcomes may not fully capture its potential therapeutic role in intensive care. Evidence from the literature suggests that albumin is a key circulating antioxidant; albumin administration improves organ function in hypoalbuminemic patients [21]. In this prospective, randomized, open-label, controlled clinical trial it was hypothesized that early albumin supplementation in septic patients with hypoalbuminemia (<30 g/L) would exert beneficial effects on oxidative stress and inflammatory responses. The primary objective of this study was to evaluate temporal changes in biomarkers directly affected by serum albumin administration, with a particular focus on markers of inflammation and oxidative stress in patients with sepsis or septic shock.

### 2.2. Study Population

Sample size was estimated based on the randomized, double-blind, controlled trial by Keshani et al. (2024) [22], which evaluated the effects of L-carnitine supplementation on inflammation, oxidative stress, and clinical outcomes in critically ill patients with sepsis. That study included 30 control and 30 L-carnitine-supplemented patients and demonstrated significant changes in C-reactive protein (CRP) levels between groups [22]. Therefore, CRP was selected as the reference parameter. The required sample size was calculated assuming a two-sided significance level (α) of 0.05 and a statistical power (1 − β) of 80%. Due to the similarity in study design and patient population, these estimates were considered appropriate for the current trial. Based on these assumptions, the minimum required sample size was estimated to be 16 patients per group.

The final study population consisted of 38 septic patients admitted to the intensive care unit (ICU) with a serum albumin level below 30 g/L. Sepsis and septic shock were defined according to the Sepsis-3 criteria and the 2021 Surviving Sepsis Campaign (SSC) guidelines. Sepsis was defined by a SOFA score increase of ≥2 points, while septic shock was defined as sepsis requiring vasopressor therapy to maintain a mean arterial pressure ≥65 mmHg, together with a serum lactate level >2 mmol/L despite adequate fluid resuscitation and in the absence of hypovolemia. The study was carried out from 4 March 2023 to 11 March 2024. Written informed consent was obtained from all participants or their next of kin prior to inclusion. Exclusion criteria included age under 18 years, pregnancy, any autoimmune disease, or lack of consent. Additional exclusions applied to patients with conditions or treatments affecting immune function, including malignant hematological diseases, chronic steroid therapy, biological therapy, immunosuppressive treatment following organ transplantation, or end-stage malignant disease. Patients were allocated to either a control group receiving standard SSC-guided pharmacological management or an albumin-supplemented group receiving albumin in addition to standard therapy. Study participants were randomized into the two groups using sealed-envelope allocation. In the intervention group patients (*n* = 18) received 3 × 100 mL of 20% albumin solution daily for three consecutive days. In the control group, albumin was not administered if serum albumin concentrations remained above 15 g/L. Control patients whose serum albumin fell below 15 g/L were excluded from the study and received albumin supplementation according to standard clinical practice.

The study was conducted in accordance with the Declaration of Helsinki. The study protocol was approved by the Ethics Committee of the University of Pécs (approval number: 7619-PTE 2023) and was registered with ClinicalTrials.gov (trial registration number: NCT03950778).

Apart from albumin administration, all pharmacological interventions were intended to be equivalent between groups, allowing for the assessment of albumin supplementation without confounding differences in medication management.

### 2.3. Standardized SSC-Guided Management in Control and Albumin-Supplemented Septic Patients at the ICU

All patients admitted to the intensive care unit with sepsis were managed according to a standardized treatment protocol based on the 2021 SSC guidelines with the aim of minimizing variability in clinical care. Management strategies were applied as uniformly as possible across the study population, regardless of group allocation. These measures included early recognition of sepsis, prompt administration of appropriate antimicrobial therapy, source control when indicated and hemodynamic support guided by established recommendations. Fluid resuscitation, vasopressor therapy, ventilatory support, renal replacement therapy, and other organ support interventions were initiated and adjusted according to the patient’s clinical condition and current best practice guidelines.

Routine monitoring, laboratory investigations, and physiological assessments were conducted following standard ICU practice and were comparable between groups. Clinical targets, including mean arterial pressure, urine output, lactate clearance, and oxygenation parameters, were managed consistently to reduce treatment bias and allow a more reliable comparison between the control and albumin-supplemented groups.

### 2.4. Medication

Patients allocated to the albumin-supplemented group received 100 mL of 20% human albumin solution (Human Albumin CSL Behring 200 g/L, 100 mL solution, CSL Behring GmbH, Marburg, Germany) three times daily for three consecutive days. The first dose was administered after study inclusion and group allocation, with subsequent doses given at approximately 8 h intervals. Albumin was administered intravenously typically over approximately 30–60 min; however no fixed infusion rate was predefined in the study protocol. During administration, patients were monitored clinically for signs of fluid overload or intolerance. No additional study-specific monitoring method was used for this purpose. Albumin administration was discontinued if clinical signs of fluid overload developed or if another clinical contraindication to continued administration was identified by the treating physician. The inclusion threshold of serum albumin below 30 g/L and the dose of 60 g of 20% albumin daily for three consecutive days were selected based on previously published evidence and clinical considerations, including the EARSS trial, which used 100 mL of 20% albumin every 8 h for three days, and the ALBIOS trial, in which up to 300 mL (60 g) of 20% albumin per day was administered according to serum albumin concentration [18,23]. These criteria were chosen to target patients with clinically relevant hypoalbuminemia while providing a standardized albumin supplementation regimen.

Pharmacological treatment was administered according to the 2021 SSC guidelines and standardized as far as clinically feasible. Empiric broad-spectrum antimicrobial therapy was initiated within 3 h of sepsis or 1 h of septic shock recognition. Antibiotic selection was guided by the suspected source of infection, local antimicrobial resistance patterns, prior microbiological data, recent antibiotic exposure, and patient-specific factors, including renal and hepatic function, etc. Blood cultures and relevant site-specific cultures were obtained prior to antibiotic administration whenever possible, without delaying therapy. Antimicrobial regimens were reviewed at least daily and de-escalated, escalated, or discontinued based on microbiological results, clinical response, and inflammatory markers. Hemodynamic support followed SSC recommendations after initial fluid resuscitation. In patients with persistent hypotension, norepinephrine was initiated as the first-line vasopressor and titrated to achieve a target mean arterial pressure (MAP) ≥65 mmHg, using invasive arterial blood pressure monitoring. Vasopressin (up to 0.03 U/min) or epinephrine was added in cases of refractory shock. Dobutamine was administered in selected patients with evidence of myocardial dysfunction or ongoing tissue hypoperfusion despite adequate MAP and intravascular volume. Intravenous hydrocortisone (typically 200 mg/day, administered as intermittent doses or continuous infusion) was considered in patients with septic shock requiring escalating or prolonged vasopressor support. Glycemic control was managed with intravenous insulin to maintain blood glucose within recommended targets, with frequent glucose monitoring to avoid hypoglycemia. Sedation and analgesia were provided using protocolized regimens, with agent selection and dosing adjusted for organ dysfunction and sedation targets. Supportive pharmacological therapies, including stress ulcer prophylaxis, venous thromboembolism prophylaxis, and renal-adjusted medication dosing, were applied consistently. Therapeutic drug monitoring was performed when indicated, particularly for antimicrobials with narrow therapeutic indices.

Sedation was performed with intravenous 2% propofol (1.5–2.5 mg/kg; Fresenius Kabi Deutschland GmbH, Bad Homburg, Germany) and sufentanil (0.0015 mg/kg; Richter Gedeon Nyrt., Budapest, Hungary), if required. Patients who required maintenance of adequate oxygenation were intubated and mechanically ventilated. For pain relief, sufentanil and acetaminophen (1 g intravenous; Fresenius Kabi Hungary Kft., Budapest, Hungary) were administered intravenously as required, according to pharmaceutical instructions.

### 2.5. Blood and Urine Sampling

Blood samples for measurement of serum albumin and biomarkers were collected five times: at baseline (Sample 1) and on each consecutive day from Day 2 to Day 5 (Samples 2–5). Initial sampling was performed immediately upon ICU admission, with subsequent collections at the same time (about 7 AM) on the following days. The kinetics of the measured parameters were evaluated over the five-day observation period.

Samples were collected through arterial catheters placed in all patients as part of routine ICU care (venous sampling was permissible when an arterial line was not accessible). However, the sampling site was not systematically documented for each individual blood draw, and the potential effect of arterial and central venous sampling on the measured biomarker concentrations was not specifically assessed and was not included in the statistical analyses. Plastic blood collection tubes with clot activator and separator gel were used for serum samples and K3EDTA-containing tubes for anticoagulated samples (Vacuette^®^, Greiner Bio-One International GmbH, Kremsmünster, Austria). Serum was used to assess total antioxidant capacity (TAC), antioxidant enzyme activities, and phenylalanine (Phe) and ortho-, meta-, and para-tyrosine (oTyr, mTyr, pTyr) concentrations. Assessment of antioxidant status and oxidative stress included measurement of reduced glutathione (GSH), plasma protein sulfhydryl (PSH) groups, myeloperoxidase (MPO) activity, catalase (CAT) activity, and serum bilirubin concentrations. Inflammatory response was assessed by measuring CRP, procalcitonin (PCT), serum concentrations of key interleukins (interleukin-1β (IL-1β), interleukin-6 (IL-6)) and by calculating the leukocyte antisedimentation rate (LAR) and the neutrophil-to-lymphocyte ratio (NLR); 24 h urine samples were used for the determination of urinary oTyr, mTyr, pTyr and Phe concentrations.

Parameters of complete blood count and LAR were determined immediately from K3EDTA-anticoagulated whole blood samples. All other samples were allowed to rest for 30–40 min before processing, followed by centrifugation (Hettich Universal 320, Andreas Hettich GmbH, Tuttlingen, Germany) at 3000× *g* rpm for 10 min (RCF = 1,497× *g*). Serum, plasma, and urine samples were aliquoted into labeled 1.5 mL reaction tubes (Sarstedt AG, Nümbrecht, Germany) and stored at −80 °C until analysis.

### 2.6. Measurement of Inflammatory and Oxidative Stress Markers

Serum TAC was quantified using a luminol-based enhanced chemiluminescence (ECL) microplate assay, according to our previously validated and reported protocol, without any modifications. The chemiluminescence signal was monitored for 10 min using a Synergy HT plate reader (BioTek Instruments Inc., Winooski, VT, USA) in kinetic mode. The TAC of the samples was determined as Trolox equivalents (TE), expressed in μM, using a calibration curve [24]. Intra-assay CVs ranged from 2.83% to 6.53% and inter-assay CVs from 3.73% to 7.87% [24]. To determine MPO and CAT activity in plasma samples, the Myeloperoxidase Assay Kit and the Catalase Assay Kit (Abbexa Ltd., Cambridge, UK) were used, respectively, according to the manufacturer’s instructions without any modifications. The intra-assay and inter-assay imprecision of the tests were <10 CV% and <12 CV%, respectively, according to the manufacturers’ specifications. Determination of reduced glutathione in plasma was performed using the Glutathione Assay Kit (Sigma-Aldrich^®^, Merck KGaA, Darmstadt, Germany). The sulfhydryl group content of plasma was assessed using Ellman’s reagent (Thermo Fisher Scientific Inc., Waltham, MA, USA). To calibrate the assay, a dilution series of L-cysteine standard (Sigma-Aldrich^®^, Merck KGaA, Darmstadt, Germany) was prepared and tested. The absorbance was measured using an EnSpire^®^ Multimode plate reader (PerkinElmer Inc., Waltham, MA, USA).

Serum IL-1β concentrations were measured using commercial enzyme-linked immunosorbent assay (ELISA) kits (Abbexa Ltd., Cambridge, UK). The intra-assay CV was reported by the manufacturer as <10% and the inter-assay CV as <12%. IL-6 was measured using a fully automated method (cobas e411 analyzer, Roche Diagnostics GmbH, Mannheim, Germany).

Complete blood count, serum CRP, PCT, total protein, and albumin levels were assessed using routine procedures at the accredited laboratory of our university (Department of Laboratory Medicine, University of Pécs Medical School, Hungary; accreditation no.: NAH-9-0008/2021).

For phenylalanine and tyrosine measurements, sample preparation involved adding 125 µL of trichloroacetic acid to 500 µL of serum or urine, followed by incubation on ice for 30 min. Precipitates were removed by centrifugation, and the resulting supernatant was filtered through a 0.2 µm membrane filter prior to analysis. Concentrations of oTyr, mTyr and pTyr, as well as Phe, were quantified using reverse-phase high-performance liquid chromatography (RP-HPLC) with fluorescence detection (tyrosines: λ_EX = 275 nm, λ_EM = 305 nm; phenylalanine: λ_EX = 258 nm).

### 2.7. Statistical Analysis

Statistical analyses were performed using R statistical software (v4.2.2; R Foundation for Statistical Computing, Vienna, Austria). Continuous variables are presented as mean with standard deviation or median with interquartile range, while categorical variables are summarized using counts and percentages.

Between-group comparisons for continuous variables were performed for each study day using appropriate statistical tests selected according to data characteristics: the Mann–Whitney U-test for continuous variables and the Chi-squared test or the Fisher test for categorical variables.

To account for repeated measurements within individuals, linear mixed-effects models were fitted for selected continuous outcomes. Models included fixed effects for study day, group, and their interaction, with subject included as a random effect, and age was included as a covariate. Model estimates are presented with corresponding 95% confidence intervals, and model assumptions were evaluated using standard diagnostic procedures. Because patients in the control group were significantly older than those in the albumin group, age adjustment was included in all primary linear mixed-effects models to account for its potential confounding effect.

Corresponding two-sided *p*-values were reported with a significance level of *p* < 0.05.

Given the exploratory nature of the analyses, post hoc comparisons across individual time points were performed without formal adjustment for multiple testing. These analyses were intended to characterize temporal patterns rather than serve as independent confirmatory hypotheses.

## 3. Results

### 3.1. Study Population and Baseline Characteristics

A total of 40 septic patients admitted to the intensive care unit were initially enrolled. During the study period, 2 patients were excluded from the final analysis: in 1 patient, computed tomography revealed multiple tumors, and 1 patient in the control group required albumin supplementation and was treated with albumin for clinical and ethical reasons on Day 3 after the serum albumin level decreased below 15 g/L. Consequently, 38 patients were included in the final analysis, of whom 18 received albumin supplementation and 20 served as controls. The number of evaluable patients decreased during the 5-day study period due to patient loss (Day 1: *n* = 18 vs. 20; Day 3: *n* = 17 vs. 17; Day 5: *n* = 15 vs. 11; albumin vs. control).

Baseline characteristics are summarized in Supplementary Table S1. Patients in the control group were significantly older (*p* = 0.026). Baseline hemodynamic variables, mechanical ventilation requirements, electrolyte levels, routine hematological parameters, renal replacement therapy requirements, and acid–base status were otherwise comparable between groups. In both groups, the most common primary infections were abdominal and respiratory, followed by soft tissue, urinary, and other sources of infection.

Most laboratory parameters and biomarker levels were assessed daily from Day 1 (baseline) to Day 5 in both groups. TAC, GSH and PSH were measured at three predefined time points during follow-up.

### 3.2. Serum Albumin and Total Protein Levels

Serum albumin levels of the two groups changed in opposite directions during the follow-up period. In the albumin-treated group, model-estimated albumin concentrations increased progressively at each successive assessment, indicating a steady and sustained rise over time. In contrast, the control group exhibited a gradual decline (Figure 1).

Baseline serum albumin values were comparable between groups. From Day 2 onward, albumin concentrations diverged and remained consistently higher in the albumin group at all subsequent time points. A statistically significant separation emerged on Days 3–5; median serum albumin concentrations with interquartile ranges in the albumin and control groups were 32.8 g/L (32.2, 35.5) vs. 24.4 g/L (22.95, 25.55) on Day 3 (*p* < 0.001); 33.85 g/L (32.4, 36.5) vs. 23.5 g/L (21.9, 26.7) on Day 4 (*p* < 0.001); and 33.0 g/L (29.45, 38.5) vs. 24.5 g/L (23.35, 27.45) on Day 5 (*p* = 0.021) (see Supplementary Figure S1).

Serum total protein also changed over time (*p* = 0.021) and showed a highly significant time–group interaction (*p* < 0.001), while the overall group effect was not significant (*p* = 0.259). In the albumin group, model-estimated total protein values increased progressively from 45.3 g/L (95% CI 41.8–48.9 g/L) on Day 1 to 52.2 g/L (95% CI 48.6–55.8 g/L) on Day 5. In contrast, the control group showed a slight decline over time, with fitted values decreasing from 46.1 g/L (95% CI 42.7–49.5 g/L) on Day 1 to 43.7 g/L (95% CI 39.9–47.1 g/L) on Day 5.

### 3.3. Inflammatory Markers

#### 3.3.1. C-Reactive Protein

Model-estimated CRP values declined significantly in both the albumin group [from 238.4 mg/L (95% CI 187.8–289.0 mg/L) to 111.5 mg/L (95% CI 58.3–164.8 mg/L)] and the control group [from 234.6 mg/L (95% CI 185.6–283.6 mg/L) to 146.4 mg/L (95% CI 91.3–201.5 mg/L)] from Day 1 to Day 5 (Figure 2), indicating parallel patterns of change throughout the study period. A significant main effect of time (*p* < 0.001) was observed. However, CRP levels did not differ significantly between groups at any time point.

#### 3.3.2. Procalcitonin

Regarding serum PCT levels, a significant negative time effect was observed (β = −4.13 per day, *p* < 0.001), indicating that PCT levels declined significantly over the 5-day observation period overall. While no significant main effect of treatment group was observed, there was a trend toward a group–time interaction, suggesting a faster decline in PCT levels in the albumin group compared with controls (*p* = 0.096). At each time point, considerable within-group variability was observed, and the confidence intervals of the two groups overlapped, showing no consistent visual separation between groups (Figure 3).

#### 3.3.3. Myeloperoxidase

Plasma MPO activity did not differ significantly between the albumin and control groups at any time point and showed no significant change over time. Descriptively, MPO levels were consistently lower in the albumin group, and no significant time–treatment interaction was observed (*p* = 0.855) (Figure 4).

#### 3.3.4. IL-1β and IL-6

Model-estimated IL-1β values did not differ significantly between the albumin and control groups (*p* = 0.819), and no significant effect of time (*p* = 0.311) or time–group interaction (*p* = 0.277) was observed. Although model-estimated IL-1β values remained stable in the albumin group, with minimal day-to-day variation [11.45 pg/mL (95% CI 7.14–15.77 pg/mL) to 11.32 pg/mL (95% CI 6.85–15.78 pg/mL)], and were numerically higher in the control group throughout follow-up [13.27 pg/mL (95% CI 9.15–17.39 pg/mL) to 17.05 pg/mL (95% CI 12.36–21.75 pg/mL)], these differences were not statistically significant (Figure 5).

Model-estimated IL-6 values decreased significantly over time in the overall cohort (*p* = 0.029). No significant group effect (*p* = 0.134) or time–group interaction (*p* = 0.171) was identified. While the fitted values suggested a more pronounced decline in the control group, this visual pattern was not statistically confirmed.

#### 3.3.5. Leukocyte-Related Inflammatory Indices

The neutrophil-to-lymphocyte ratio decreased significantly over time in the overall cohort (*p* = 0.006). Fitted NLR values declined progressively in both the albumin group [from 22.40 (95% CI 14.98–29.83) to 15.58 (95% CI 7.91–23.25)] and the control group [from 21.53 (95% CI 14.40–28.67) to 13.58 (95% CI 5.68–21.49)] from Day 1 to Day 5. However, no significant difference was observed between the albumin and control groups (*p* = 0.922), and the time–group interaction was not significant (*p* = 0.830) (see Supplementary Figure S3).

Based on the linear mixed model, the upper fraction of the LAR showed a non-significant tendency toward lower values over time (*p* = 0.069), without significant effects of treatment group (*p* = 0.542) or time–group interaction (*p* = 0.519) (Supplementary Figure S4). The lower LAR fraction showed no significant change over time (*p* = 0.457) and did not differ between groups (*p* = 0.515). No significant time–group interaction was detected (*p* = 0.460) (see Supplementary Figure S5).

### 3.4. Oxidative Stress and Antioxidant Parameters

#### 3.4.1. Total Serum Antioxidant Capacity

Changes in serum TAC over the study period for both groups are shown in Figure 6.

Based on the linear mixed model, serum TAC levels showed a significant decline over time in both groups, and the temporal trajectories remained broadly parallel. Model-estimated TAC values were consistently higher in the albumin group at all measured time points: on Day 1, 535.4 µmol/L (95% CI 447.4–623.4) versus 391.2 µmol/L (95% CI 307.9–474.5); on Day 3, 481.6 µmol/L (95% CI 402.4–560.8) versus 313.5 µmol/L (95% CI 236.3–390.6); and on Day 5, 427.7 µmol/L (95% CI 337.1–518.3) versus 235.8 µmol/L (95% CI 140.7–330.8) in the albumin and control groups, respectively.

In exploratory between-group comparisons, TAC was higher in the albumin group on Day 3 (*p* = 0.045) and Day 5 (*p* = 0.004), whereas baseline values did not differ significantly (*p* = 0.167) (Figure 7). Despite these day-specific differences, no significant time–group interaction was observed.

#### 3.4.2. Glutathione

Model-estimated glutathione values showed a significant group–time interaction (*p* < 0.001) (Figure 8). In the albumin group, model-estimated GSH values increased from 74.11 µmol/L (95% CI 60.16–88.06 µmol/L) on Day 1 to 116.96 µmol/L (95% CI 102.33–131.59 µmol/L) on Day 5, whereas in the control group they decreased from 90.15 µmol/L (95% CI 76.89–103.40 µmol/L) to 76.37 µmol/L (95% CI 60.10–92.65 µmol/L). Accordingly, GSH values were higher in the control group at baseline and became higher in the albumin group from Day 3 onward, with progressive separation between groups until Day 5 (Figure 9).

#### 3.4.3. Plasma Protein Sulfhydryl Groups

PSH levels followed divergent patterns between groups over the observation period. In the albumin group, model-estimated PSH values increased progressively over time, rising from 0.313 mmol/L (95% CI 0.226–0.400 mmol/L) on Day 1 to 0.487 mmol/L (95% CI 0.398–0.576 mmol/L) on Day 5. In contrast, the control group showed a slight decline over time, with fitted values decreasing from 0.342 mmol/L (95% CI 0.260–0.424 mmol/L) on Day 1 to 0.314 mmol/L (95% CI 0.221–0.408 mmol/L) on Day 5. Consequently, PSH was initially lower in the albumin group, became higher from the second day onward, with the between-group difference widening over subsequent days (Figure 10).

A significant time–group interaction was observed, reflecting divergent temporal patterns between groups, with PSH levels consistently higher in albumin-treated patients throughout follow-up. Measured PSH levels demonstrated a significant difference between the two groups on Days 3 and 5 (*p* = 0.004) (Figure 11).

#### 3.4.4. Catalase Activity

CAT activity did not change significantly over the 5-day study period (*p* = 0.161) and did not differ significantly between the albumin and control groups (*p* = 0.753). Model-estimated CAT values remained relatively stable in the albumin group (Day 1: 64.6 U/mL, 95% CI 39.5–89.7 U/mL; Day 5: 62.1 U/mL, 95% CI 36.4–87.8 U/mL), whereas lower fitted values were observed in the control group at later time points (Day 1: 66.8 U/mL, 95% CI 43.0–90.5 U/mL; Day 5: 48.0 U/mL, 95% CI 21.6–74.4 U/mL). However, the confidence intervals overlapped substantially throughout follow-up. No significant time–group interaction was observed (*p* = 0.285), indicating that the temporal pattern of CAT activity was not significantly modified by albumin supplementation (Figure 12).

#### 3.4.5. Phenylalanine and Ortho-, Meta-, and Para-Tyrosine Levels in Serum and Urine

Serum and urinary tyrosine isomers were analyzed as exploratory markers of hydroxyl radical-mediated phenylalanine oxidation, while pTyr was evaluated as the physiological tyrosine isomer. Estimated serum oTyr and mTyr values changed significantly over the 5-day study period, with serum oTyr showing an increasing trajectory (albumin-treated group—Day 1: 411.2 nmol/L, 95% CI −28.0–850.4 and Day 5: 965.5 nmol/L, 95% CI 518.0–1413.1; control: Day 1: 246.2 nmol/L, 95% CI −167.0–659.6 and Day 5: 357.7 nmol/L, 95% CI −97.8–813.3) and serum mTyr a decreasing trajectory (albumin-treated group—Day 1: 114.8 nmol/L, 95% CI 66.5–163.0 and Day 5: 66.9 nmol/L, 95% CI 17.9–115.8; control: Day 1: 51.3 nmol/L, 95% CI 5.9–96.8 and Day 5: 30.1 nmol/L, 95% CI −18.7–79.1); however, no significant albumin-specific effect was detected. Serum pTyr showed no significant change during follow-up. In contrast, urinary oTyr and mTyr demonstrated significant time–group interactions (*p* = 0.004 and *p* = 0.001 respectively). Urinary pTyr concentration increased significantly over the observation period (*p* = 0.002), without a significant group-related effect. The concentration of oTyr and mTyr isomers displayed increasing kinetics in the albumin-treated group, while the control group showed a decreasing trajectory for both oTyr and mTyr levels (Figure 13).

To account for variations in urine dilution and daily urine, 24 h urinary excretion was calculated to provide a more reliable estimate of the total amount of each tyrosine isomer eliminated during the observation period. 24 h pTyr excretion showed a significant time–group interaction (*p* = 0.013). Model-estimated values remained relatively stable in the albumin-treated group, increasing from 34.27 µmol/24 h on Day 1 to 39.95 µmol/24 h on Day 5, whereas a progressive increase from 62.25 µmol/24 h to 126.32 µmol/24 h was observed in the control group. mTyr excretion showed a decreasing trajectory in both groups, which was numerically more pronounced in controls. oTyr excretion showed divergent trajectories between groups, with a significant time–group interaction (*p* = 0.046). Model-estimated values increased from 16,178.20 nmol/24 h on Day 1 to 49,832.30 nmol/24 on Day 5 in the albumin-treated group, whereas they decreased from 14,831.17 nmol/24 to 8880.02 nmol/24 in controls.

To characterize the relative urinary balance of physiological and oxidative tyrosine isomers, we additionally analyzed tyrosine isomer-to-phenylalanine and abnormal-to-physiological tyrosine isomer ratios. The urinary pTyr/Phe ratio showed a significant time–group interaction (*p* = 0.033), remaining relatively stable in the albumin-treated group while increasing progressively in controls. The urinary mTyr/pTyr ratio also showed a significant interaction (*p* = 0.038), with a slight increase in the albumin-treated group and a marked decrease in controls. No significant interactions were observed for urinary mTyr/Phe, oTyr/Phe, or oTyr/pTyr. Similarly, none of the serum ratio measures demonstrated an albumin-specific effect.

These findings indicated that the oxidative tyrosine isomers followed unique temporal patterns, with the clearest group-related differences observed in urine rather than in serum. Regarding serum and urinary phenylalanine concentrations, no significant time–group interactions were found.

Detailed linear mixed-effects model results are presented in Supplementary Tables S2 and S3. Additional analyses of 5-day and 30 day mortality are provided in Supplementary Table S4.

## 4. Discussion

This study assessed the effects of a 3-day 3 × 100 mL albumin (20%) administration on oxidative stress and inflammatory markers in ICU patients with sepsis and hypoalbuminemia. Our results show a clear divergence in serum albumin concentrations between the two groups over time. Although baseline levels were identical, albumin concentrations increased progressively in patients receiving supplementation, while a gradual decline was observed in the control group. This trend is consistent with the development of hypoalbuminemia during acute illness, which is associated with systemic inflammation, increased capillary permeability, dilutional effects of fluid therapy, and reduced hepatic synthesis [15,25,26,27]. In this context, exogenous albumin (20%) administration appears to partially counteract these processes and help maintain circulating albumin levels. A statistically significant increase in serum albumin concentrations was observed from Day 3 onward, suggesting that several days of administration may be required to achieve a sustained intravascular effect. This delayed response likely reflects the dynamic distribution of albumin between the intravascular and interstitial compartments, particularly under conditions of increased vascular permeability. The progressively widening difference between the groups also indicates a cumulative effect of repeated albumin administration. These observations are in line with findings from the SAFE Study and the ALBIOS Trial, which also reported higher circulating albumin levels following albumin therapy [18,28]. However, the ATTIRE Trial demonstrated that correcting hypoalbuminemia does not necessarily improve clinical outcomes, highlighting the complex role of albumin in critical illness [29]. Our findings confirm that albumin supplementation effectively increases serum albumin concentrations over time, while untreated patients tend to show a progressive decline, although the clinical significance of this biochemical improvement remains uncertain.

Sepsis is an inflammatory process accompanied by increased levels of inflammatory parameters, e.g., CRP, PCT, and interleukins [30,31,32]. Both inflammatory markers (CRP and PCT) showed a similar pattern over the observation period. CRP levels decreased significantly in both groups, reflecting the gradual resolution of systemic inflammation. No significant differences were observed between the albumin and control groups. The parallel decline suggests that albumin supplementation did not substantially influence CRP dynamics beyond the effect of standard clinical management. PCT showed a comparable trend. PCT concentrations decreased over time in both groups, which is consistent with the expected course during recovery from infection or systemic inflammatory states. The similar downward trends observed in both groups suggest that the measured parameter decreased over time regardless of albumin treatment. There is no evidence that albumin supplementation influenced this change. The reduction likely reflects the underlying clinical improvement and treatment effectiveness common to all patients during the study period.

Serum IL-1β levels remained relatively stable in the albumin group throughout the five-day observation period, whereas a gradual increase was observed in the control group, although with considerable variability within the group. While these differences were not statistically significant, this divergence may suggest that albumin supplementation may help modulate the inflammatory response by slowing the rise in the pro-inflammatory cytokine IL-1β. Nevertheless, the differences observed are modest and should be interpreted cautiously. These findings are consistent with previous reports suggesting that albumin has anti-inflammatory properties, potentially through endothelial stabilization, modulation of cytokine release, and reduction in oxidative stress [33,34,35]. However, based on the present data, no definitive conclusion can be drawn regarding a direct effect of albumin on IL-1β-mediated inflammatory pathways.

Serum IL-6 levels declined over the five-day period in both the albumin and control groups. This might be attributed to the fact of general reduction in systemic inflammation. Although the control group showed higher IL-6 levels early during follow-up and greater variability over time, the between-group differences were not statistically significant. Therefore, these findings suggest that albumin supplementation might not have an effect on systemic cytokine signaling and IL-6 kinetics.

MPO is released by neutrophils in response to inflammatory stimuli [36,37,38]. Plasma MPO increased progressively in both albumin and control groups, with no significant between-group differences at any time point. Although MPO levels were consistently higher in controls and the absolute difference widened modestly over time, these differences remained non-significant, and the broadly parallel temporal changes suggest that early MPO dynamics were primarily driven by shared inflammatory processes rather than by albumin-related modulation. The observed antioxidant effects (higher TAC, GSH, and PSH in the albumin group) therefore likely reflect albumin’s ability to buffer the oxidative consequences of MPO-derived reactive species, neutralizing hypochlorous acid and other oxidants after their formation, rather than preventing MPO release itself [39,40]. The absence of a significant effect on MPO may indicate that albumin supplementation did not impair innate immune activation in septic patients.

Albumin, the most abundant protein in plasma, performs several key antioxidant roles and has a major role in serum TAC [7,41]. It scavenges free radicals through its Cys-34 thiol, binds and neutralizes redox-active metals and lipid peroxidation products, and acts as a sacrificial target to shield other proteins and lipids from oxidative damage [42]. Under conditions of systemic inflammation and oxidative stress, circulating albumin can become oxidized, reducing its antioxidant capacity. Supplementing with exogenous albumin may help restore extracellular antioxidant defenses, limit oxidative tissue injury, and influence inflammatory signaling [10]. However, the redox characteristics of therapeutic HSA preparations may vary according to the manufacturing process. Mori et al. evaluated three lots of Human Albumin 20% CSL-Behring, the same commercial preparation used in the present study, and reported a free Cys34 fraction of approximately 53% by LC-ESI-MS and 31% by the DTNB free-thiol assay. Although lower than the physiological free-thiol fraction of circulating HSA, the preparation retained measurable radical-scavenging activity [43]. Nonetheless, the redox status of the specific batches administered in our study was not determined; these published values cannot be directly extrapolated to our patients.

In this study, serum TAC declined over the 5-day observation period in both groups. Baseline antioxidant capacity did not differ significantly between the groups (*p* = 0.167); however, a significant difference was observed by Day 3 (*p* = 0.045) and became more pronounced by Day 5 (*p* = 0.004). Furthermore, the progressive divergence between groups from Day 3 to 5 suggests a cumulative effect, with albumin-treated patients maintaining higher TAC levels over time. This trend suggests that albumin supplementation may help preserve systemic antioxidant capacity–or slow its decline–during the early period of treatment.

Sepsis and inflammation are characterized by excessive production of ROS and RNS, driven by both dysregulated inflammation and mitochondrial dysfunction, which together create significant oxidative stress [44]. Glutathione is a key intracellular antioxidant that maintains redox balance by scavenging ROS/RNS and serving as a reducing substrate for enzymes such as glutathione peroxidases, limiting oxidative damage to lipids, proteins, and DNA [45,46]. Through its reversible oxidation to glutathione disulfide (GSSG) and subsequent enzymatic regeneration, GSH helps buffer oxidative stress and preserve protein thiols. During prolonged oxidative stress, GSH may be converted to GSSG faster than it is regenerated, leading to a decline in reduced GSH and a more oxidizing cellular environment. In this setting, albumin, mainly through its Cys-34 thiol and its capacity to bind reactive molecules, may serve as an extracellular antioxidant. In the present study, plasma GSH concentration followed divergent patterns between groups: levels increased steadily in the albumin group from Day 1 to 5, while they gradually declined in controls. Although initially higher in controls at baseline, the trends crossed, and levels remained higher in the albumin group from Days 3 to 5. These findings may indicate better preservation of the circulating reduced glutathione pool in patients receiving albumin, whereas the decline in controls may be consistent with continued oxidative stress and reduced availability of circulating GSH. However, the underlying mechanism cannot be established from the present data, as GSSG concentrations, the GSH/GSSG ratio and glutathione-related enzyme activity were not assessed.

PSH, largely represented by free cysteine residues on circulating proteins, are particularly vulnerable to oxidation by ROS and RNS. Because of this susceptibility, they are commonly used as markers of systemic oxidative stress. PSH is an antioxidant that, together with GSH, other thiols, and various non-enzymatic and enzymatic factors, may regulate intracellular metabolism and protect biological structures and functions from damage caused by electrophiles and oxygen-derived species [47]. The gradual increase in PSH observed in the albumin-treated group can be explained by several biological mechanisms. Administration of exogenous albumin introduces additional reduced thiol groups into the circulation, since each albumin molecule contains a free Cys-34 residue that contributes to the overall PSH pool. Albumin may also act as a preferential target for oxidative reactions, thereby shielding other circulating proteins from thiol oxidation. Furthermore, its ability to bind redox-active metals, lipid peroxidation products, and other pro-oxidant molecules may reduce oxidative pressure in the extracellular environment. Together, these effects could help maintain protein thiol groups in their reduced form [48]. The steady increase in PSH in the albumin group, in contrast to the decline seen in controls, is therefore consistent with a cumulative protective effect that becomes more apparent with continued albumin administration over the 5-day observation period.

CAT is a widely distributed antioxidant enzyme that converts hydrogen peroxide into water and oxygen, playing a key role in cellular protection against oxidative stress [49,50,51]. In the present study, CAT activity was not significantly modified by albumin supplementation, although the fitted trajectories suggested a slower decline in CAT activity in the albumin group. Several biological mechanisms can contribute to this trend. Albumin’s antioxidant actions may reduce extracellular oxidative stress, lowering the intracellular demand for enzymes like CAT. By buffering reactive oxygen species outside the cell, albumin could help preserve CAT from overuse or oxidative inactivation, allowing its activity to remain relatively stable. Additionally, albumin may support CAT indirectly by modulating inflammatory pathways or by protecting the enzyme from oxidative modifications. CAT activity in the albumin group declined modestly with little day-to-day variation and remained near baseline, whereas the control group showed a more pronounced, progressive reduction over the consecutive days.

oTyr, mTyr and pTyr are structural isomers derived from Phe, with pTyr being the physiological form produced enzymatically via phenylalanine hydroxylase [52]. Under oxidative stress, hydroxyl-radical attack on Phe can generate all three tyrosine isomers in variable ratios. While pTyr is formed mostly physiologically in enzymatic pathways, and only in small amounts through oxidative mechanisms, the other two isomers (oTyr and mTyr) are the result of reactive oxygen species-mediated formation and are often described as pathological or abnormal isomers. These abnormal tyrosine isomers, previously used only as biochemical markers of oxidative stress, are potential mediators of oxidative stress damage, showing toxicity to cells via mechanisms like incorporation into proteins, induction of apoptosis, and inhibition of cell proliferation, and the accumulation of these isomers could contribute to disease pathogenesis [53,54].

In line with previous observations, the three tyrosine isomers showed complex and non-uniform kinetics in sepsis; similarly, in our cohort, serum pTyr, mTyr, and oTyr did not follow a common temporal pattern [55]. We found no significant change in serum pTyr, but an increasing trajectory of serum oTyr and a decreasing trajectory of serum mTyr. The ratio of tyrosine isomer formation via hydroxyl-radical-mediated phenylalanine hydroxylation depends on the oxidant system and the surrounding chemical environment, with several in vitro studies suggesting a slight general preference for oTyr formation [53]. No significant albumin-specific divergence was seen in serum tyrosine levels.

The urinary findings were more complex and depended on whether concentrations, 24 h excretion, or ratio-based indices were considered. Urinary oTyr and mTyr concentrations showed significant time–group interactions. In the control group, consistent with findings from a previous pilot study, urinary oTyr and mTyr showed declining trajectories after an early peak [55]. By contrast, the albumin-treated group showed an increasing concentration of these oxidative isomers. The 24 h excretion analyses partly corroborated, but also refined, the findings obtained from urinary concentration measurements. oTyr excretion similarly showed an increasing trajectory in albumin-treated patients. In contrast, mTyr excretion declined over time in both groups, although the decrease was numerically less pronounced in the albumin-treated group. Nevertheless, the urinary mTyr/pTyr ratio showed a significant albumin-specific increase, indicating a differential change in the relative balance between oxidative and physiological tyrosine isomers. Urinary pTyr concentrations increased over time irrespective of group allocation; however, a progressive increase in 24 h urinary pTyr excretion and urinary pTyr/Phe ratio was seen in controls, with stable trajectories observed in albumin-treated patients.

Oxidative mTyr and oTyr have been reported to exhibit higher renal fractional excretion, suggesting preferential tubular reabsorption of pTyr [54]. Ipson et al. proposed that the clearance of pathological tyrosine isomers may represent a protective mechanism against oxidative stress [53]. Our findings, partly compatible with this hypothesis, suggest that albumin supplementation was associated with an altered, isomer-specific urinary tyrosine profile, characterized by a more stable physiological tyrosine-handling pattern and promoting the elimination of the potentially harmful oTyr isomer. However, because fractional excretion could not be calculated, the present results do not establish enhanced renal clearance, preserved tubular reabsorption, or a direct renal mechanism.

This study provides preliminary evidence suggesting that albumin supplementation is associated with preservation of systemic antioxidant defenses and attenuation of oxidative stress in critically ill patients. Across multiple complementary biomarkers, albumin-treated patients demonstrated a coordinated pattern of antioxidant preservation that became progressively more evident over the observation period. Our findings suggest that albumin primarily supports extracellular antioxidant defenses and redox balance rather than directly modulating systemic inflammation or neutrophil activation. The progressive divergence between groups over the 5-day study period suggests a cumulative protective effect: sustained albumin exposure helps maintain or restore antioxidant reserves, while patients in the control group exhibit progressive depletion under unmitigated oxidative stress.

### Clinical Implications and Future Directions

The consistent antioxidant-preserving effects of albumin across multiple biomarkers suggest it may help mitigate oxidative stress in critically ill patients. However, the clinical significance of these biochemical changes, such as potential reductions in organ dysfunction, ICU stay, or mortality, remains uncertain and requires evaluation in adequately powered studies with clinical endpoints. Key questions also include the optimal dosing, timing, and duration of albumin therapy to maximize antioxidant benefits. Further investigation is needed regarding the mechanisms linking albumin to preservation of GSH and CAT, including measures of oxidative damage and intracellular redox signaling. The lack of effects on CRP, PCT, and MPO indicates that albumin’s antioxidant activity may be largely independent of upstream inflammatory modulation, and future studies should explore its impact on oxidative injury in isolation from systemic inflammation.

Our findings should be interpreted considering several limitations. The sample-size calculation was based on a previous L-carnitine study, which may not accurately reflect the expected effects and variability of albumin supplementation. In addition, both the intervention and the blinding design of the L-carnitine study differed from those of the present study. Therefore, the resulting sample-size estimate should be regarded as a pragmatic approximation rather than an outcome-specific power calculation for albumin supplementation. The evaluation of multiple outcomes and comparisons across study days and biomarkers may have increased the risk of type I error; therefore, significant findings should be interpreted cautiously. The primary conclusions of the study were based on mixed-effects models evaluating longitudinal treatment effects over time, whereas daily comparisons were performed as secondary exploratory analyses to facilitate interpretation of temporal patterns. Although the RP-HPLC method used for tyrosine-isomer measurements was an already established procedure described and repeatedly applied by the same laboratory, study-specific intra-assay and inter-assay coefficients of variation were not available, limiting a complete assessment of analytical precision in the present dataset.

Participant numbers declined over time, reducing statistical power and introducing the possibility of selection bias at later time points. For some biomarkers, including MPO, GSH, PSH, and CAT, sample sizes were very small, limiting the strength and generalizability of the conclusions. The study also did not account for baseline differences or clinical severity, which could have influenced the results. Furthermore, while albumin appears to preserve antioxidant defenses, the exact mechanisms remain unclear, and we cannot confirm a direct cause-and-effect relationship. Urinary creatinine concentrations were not available; therefore, fractional excretion of the tyrosine isomers could not be calculated. Although 24 h urinary excretion and several tyrosine isomer-to-phenylalanine and abnormal-to-physiological tyrosine-isomer ratios were analyzed, these measures do not account for glomerular filtration or differential tubular handling. Adjustment for serum creatinine was explored in mixed-effects models; however, due to the limited sample size and number of covariates, the resulting models were unstable and were not considered reliable for inference. Consequently, changes in renal function or acute kidney injury may have contributed to the observed urinary tyrosine-isomer patterns. The redox status of the administered HSA was not assessed, and therefore its potential contribution to the observed changes in antioxidant capacity cannot be fully determined. Attrition during the 5-day observation period, mainly due to ICU discharge or death, resulted in unequal follow-up and a potential risk of survivor bias, particularly for Day 5 outcomes. Fluid balances were not systematically assessed and may have influenced biomarker concentrations.

## 5. Conclusions

Albumin supplementation may help preserve systemic antioxidant defenses during oxidative stress by maintaining TAC, GSH, and PSH. By increasing circulating albumin, extracellular antioxidant buffering is enhanced, thiol depletion is limited, and enzymatic antioxidants are shielded from inactivation. Albumin’s primary benefit lies in protecting the body’s antioxidant systems rather than directly modulating inflammatory processes. Clinically, this antioxidant-preserving effect may help maintain tissue integrity during systemic inflammation and oxidative stress, highlighting a potential therapeutic role for albumin beyond fluid replacement. Our findings demonstrate an association with improved systemic redox status rather than confirmed clinical benefit. Further adequately powered studies incorporating clinical endpoints and longer follow-up are required to determine whether preservation of antioxidant defenses translates into meaningful improvements in organ function and patient outcomes. Future studies should explore how correcting albumin levels impacts meaningful clinical outcomes and should elucidate the mechanisms by which albumin exerts its antioxidant effects in critically ill patients.

## Figures and Tables

**Figure 1:**
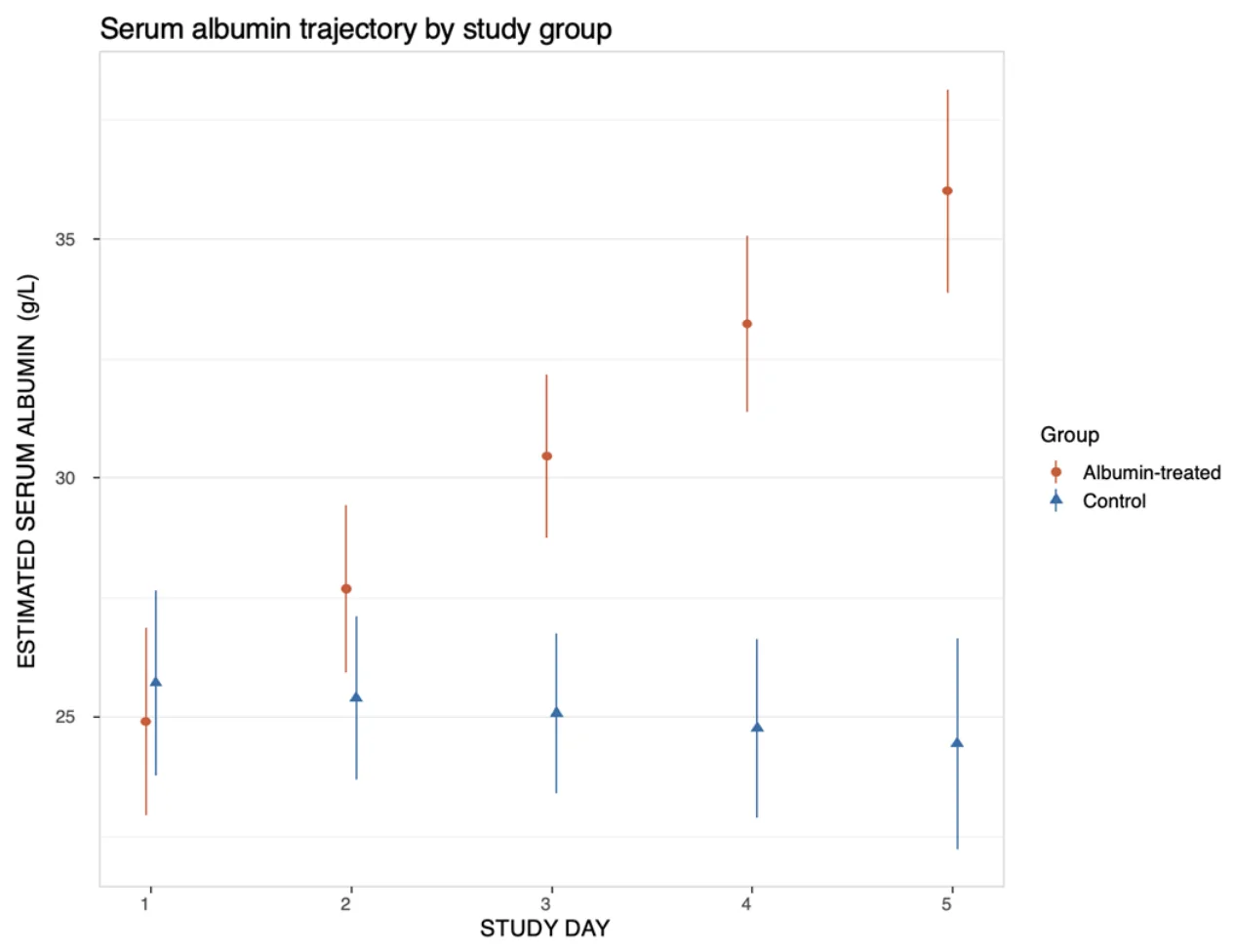
Model-estimated serum albumin concentrations (with 95% confidence intervals) over 5 days, derived from linear mixed-effects modeling in the albumin-treated and control groups, are shown. Group–time interaction from the linear mixed-effects model: *p* < 0.001.

**Figure 2:**
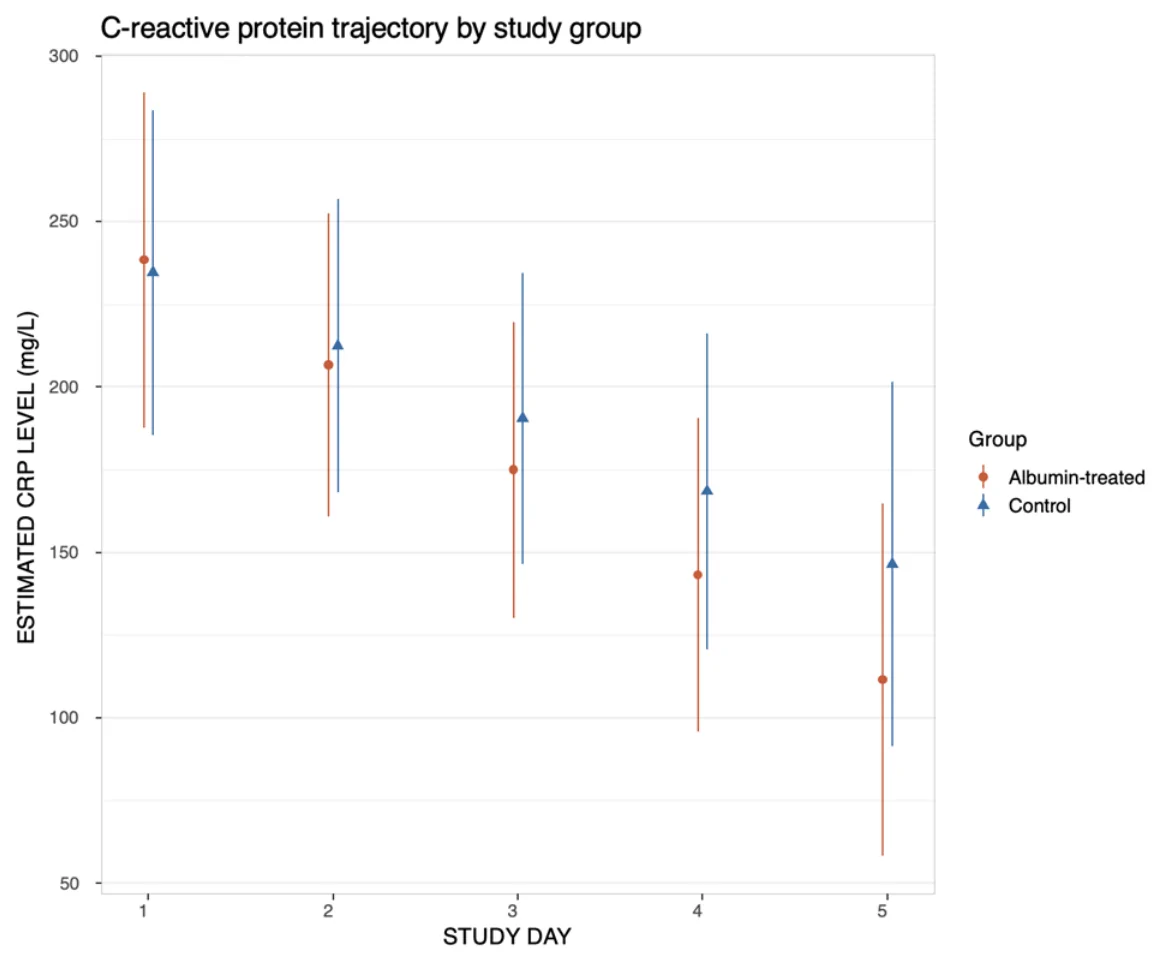
Model-estimated C-reactive protein (CRP) over the 5-day study period in the albumin-treated and control groups. Points represent fitted values from the linear mixed-effects model, and error bars indicate 95% confidence intervals. A significant main effect of time (*p* < 0.001) was observed, whereas the group × time interaction was not significant (*p* = 0.324).

**Figure 3:**
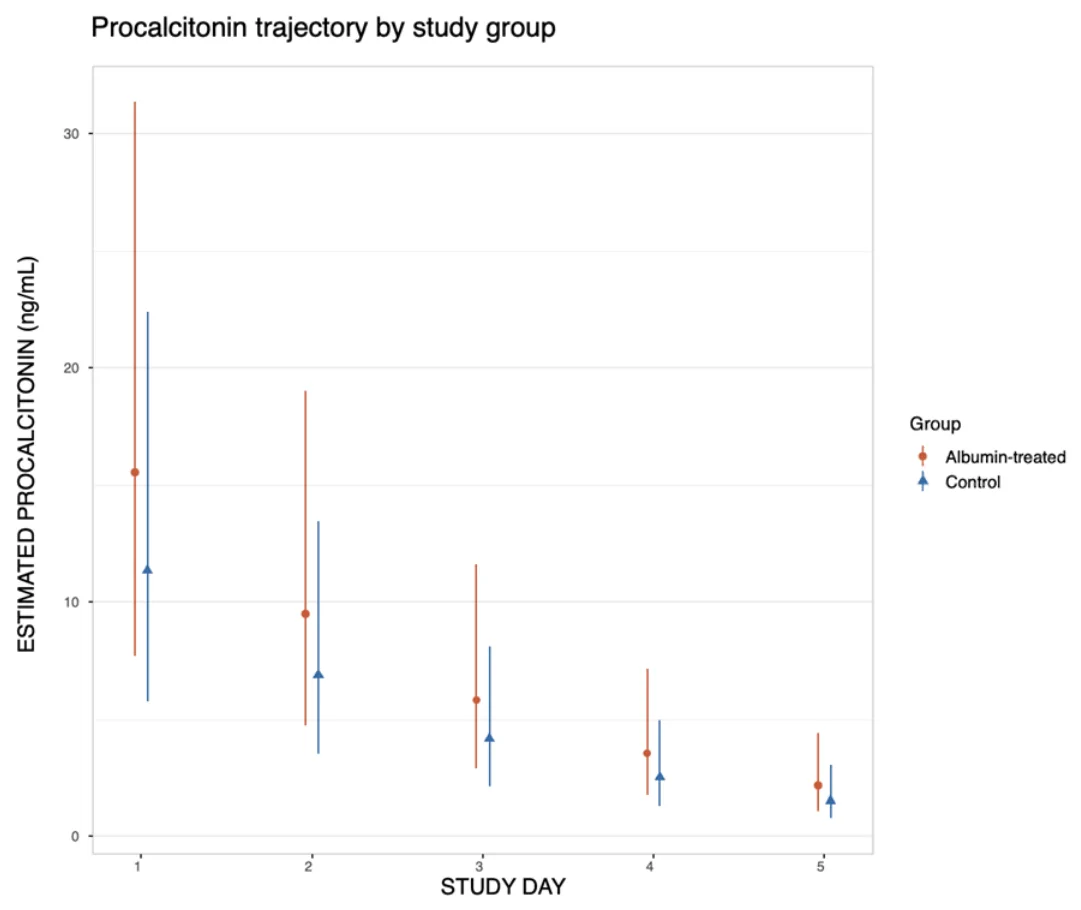
Effect of 5-day albumin administration on serum procalcitonin (PCT): control vs. albumin groups (Days 1–5). Points represent fitted values from a linear mixed-effects model, with error bars indicating 95% confidence intervals. A significant main effect of time (*p* < 0.001) was observed, whereas the group × time interaction was not significant (*p* = 0.096).

**Figure 4:**
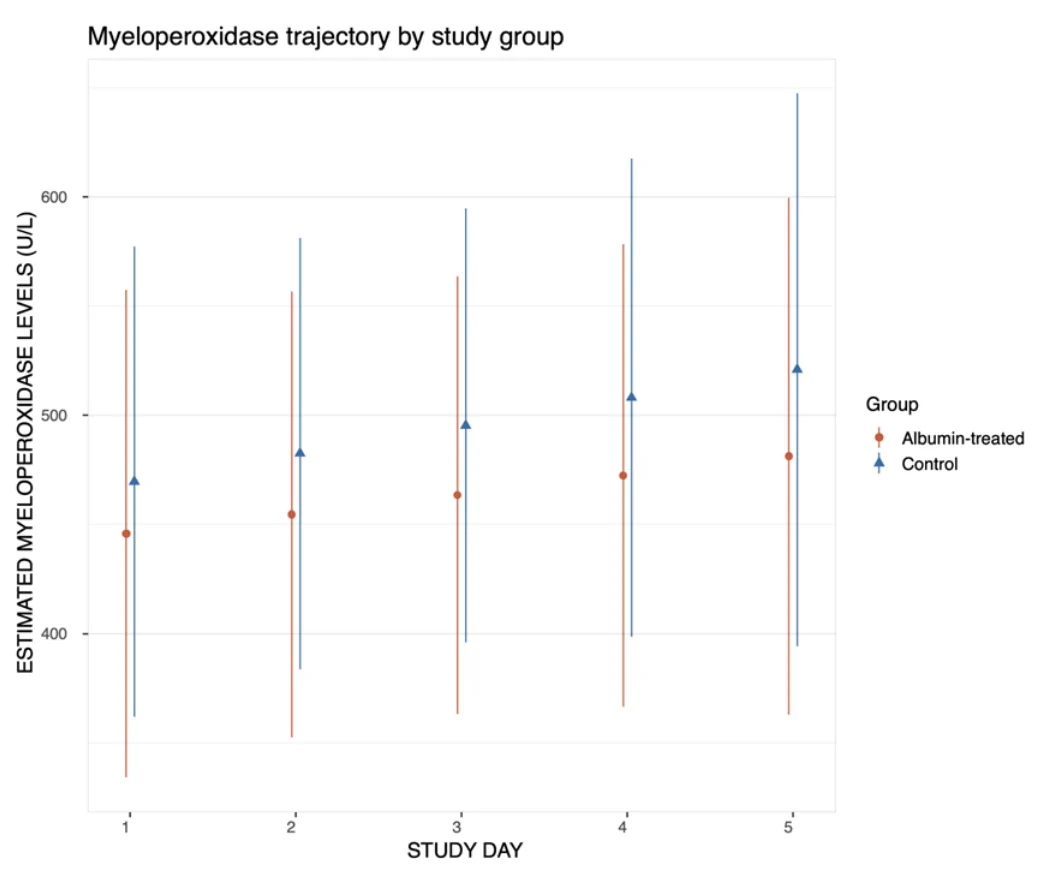
Effect of 5-day albumin administration on plasma myeloperoxidase (MPO) in control and albumin-treated groups (Days 1–5). Points represent fitted values from the linear mixed-effects model, and error bars indicate 95% confidence intervals. No significant time × group interaction was observed (*p* = 0.855).

**Figure 5:**
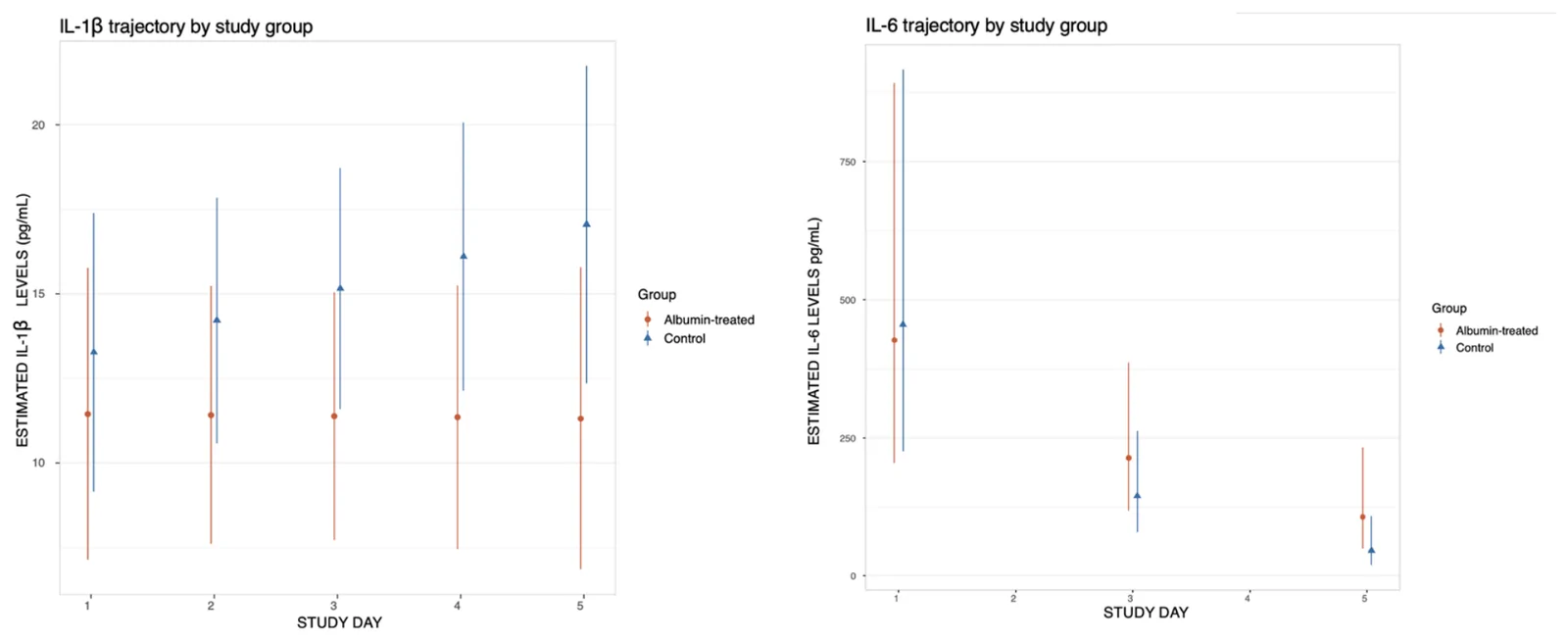
Effect of 5-day albumin administration on interleukin-1β and interleukin-6 (IL-1β, IL-6) levels in control and albumin-treated groups. Points represent fitted values from the linear mixed-effects model, and error bars indicate 95% confidence intervals. A significant main effect of time was observed in interleukin-6 levels (*p* = 0.029), whereas the time–group interaction was not significant (IL-1β, *p* = 0.277; IL-6, *p* = 0.171).

**Figure 6:**
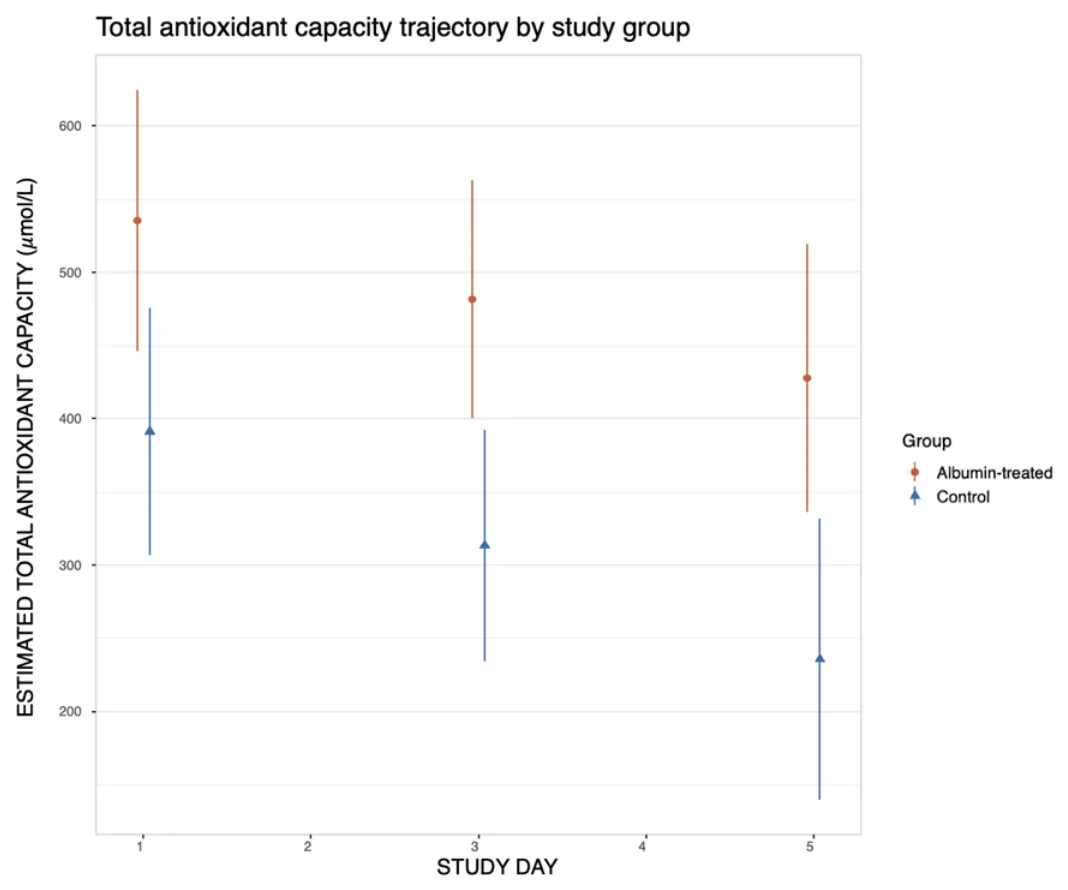
Estimated serum total antioxidant capacity (TAC) in the albumin-treated and control groups on Days 1, 3 and 5. Points represent fitted values from the linear mixed-effects model, and error bars indicate 95% confidence intervals. A significant main effect of time (*p* = 0.011) was observed, whereas the group × time interaction was not significant (*p* = 0.441).

**Figure 7:**
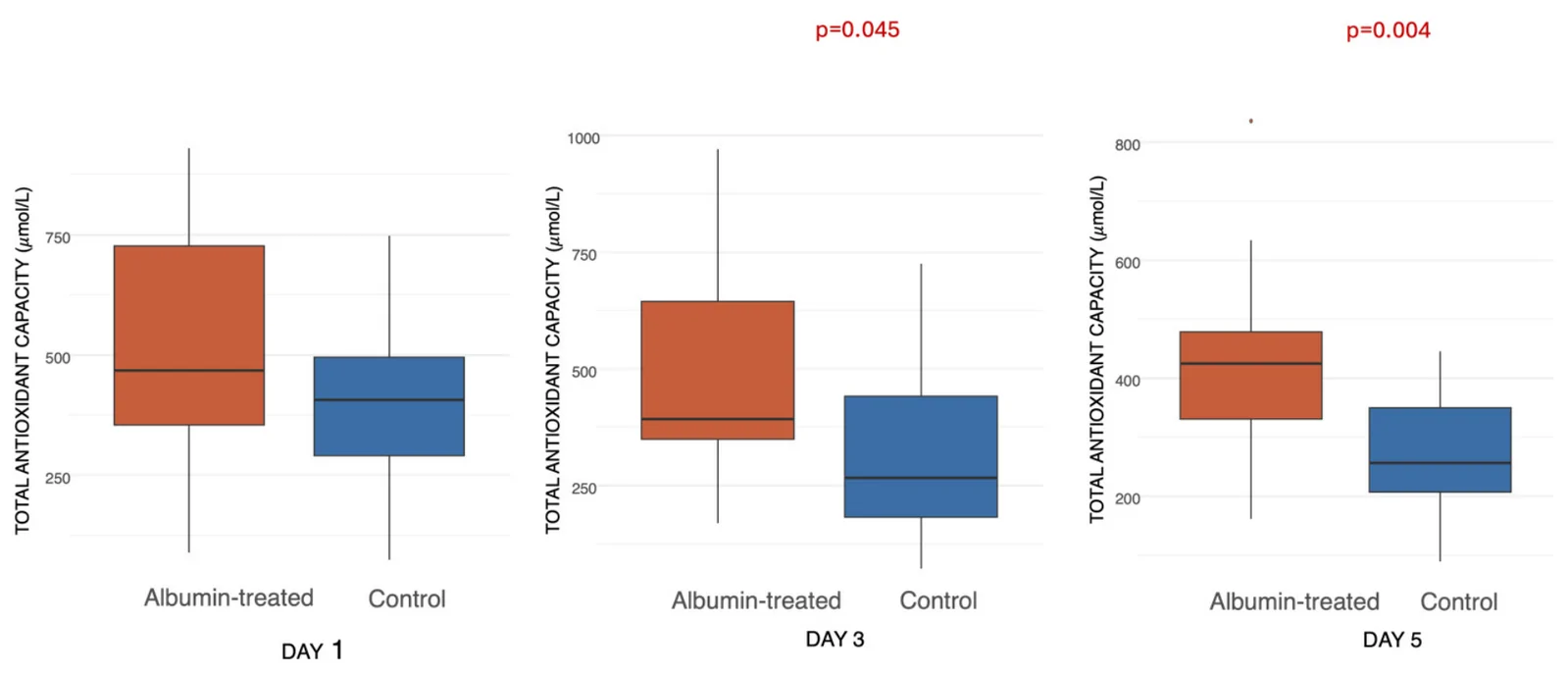
Changes in serum total antioxidant capacity (TAC) from baseline to Day 5 in control and albumin-treated groups. Data are presented as boxplots showing the median, interquartile range, and range, with outliers displayed as individual points. TAC levels demonstrated a significant difference between the two groups on Days 3 and 5 (Day 1 *p* = 0.167, Day 3 *p* = 0.045, Day 5 *p* = 0.004). Between-group comparisons at each time point were performed using the Wilcoxon rank-sum exact test.

**Figure 8:**
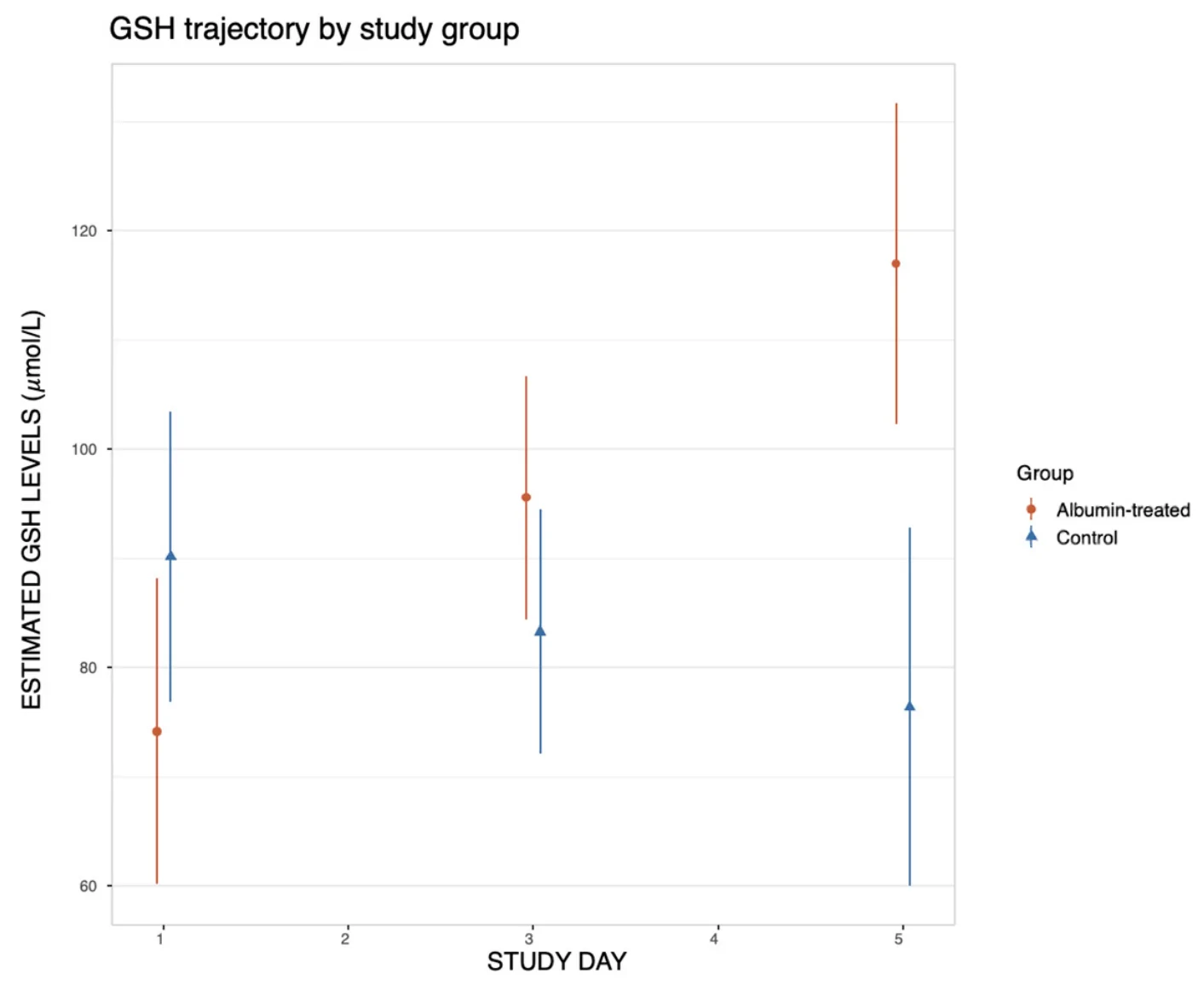
Model-estimated plasma glutathione (GSH) levels in the albumin-treated and control groups (Days 1, 3, and 5). GSH levels increased over time in the albumin group, while declining in the control group. Points represent fitted values from the linear mixed-effects model, and error bars indicate 95% confidence intervals. Group × time interaction from the linear mixed-effects model: *p* < 0.001.

**Figure 9:**
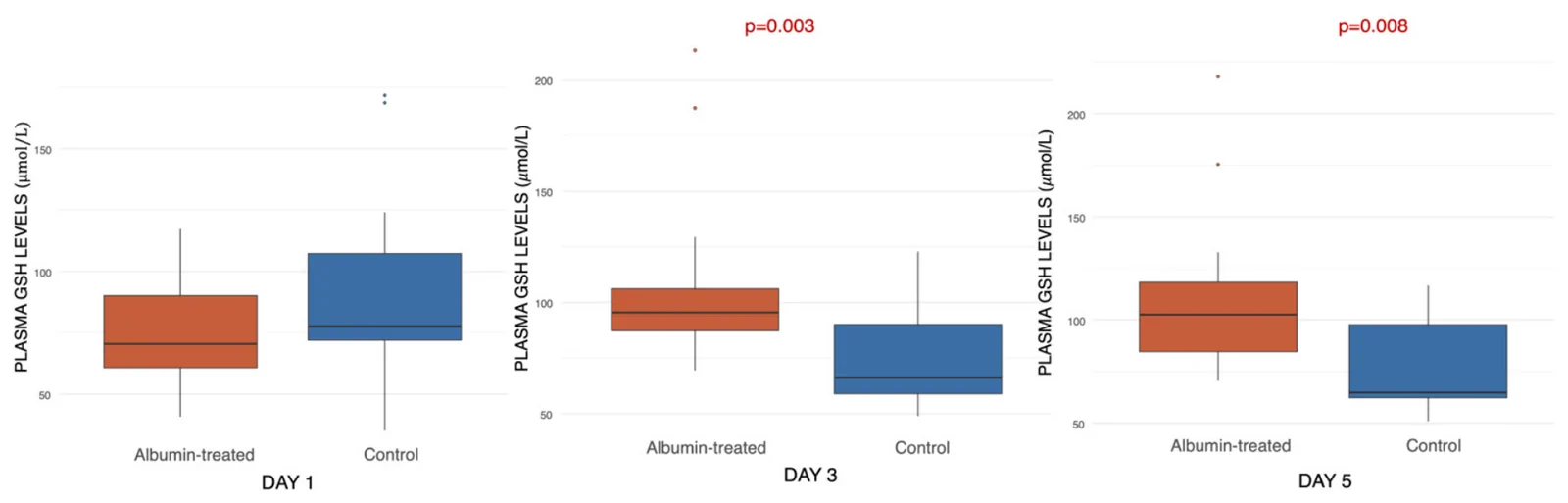
Change in plasma glutathione (GSH) Levels from baseline to Day 5 in the albumin-treated and control groups. Data are presented as boxplots showing the median, interquartile range, and range, with outliers displayed as individual points. GSH levels demonstrated a significant difference between the two groups on Days 3 and 5 (Day 1 *p* = 0.112, Day 3 *p* = 0.003, Day 5 *p* = 0.008). Between-group comparisons at each time point were performed using the Wilcoxon rank-sum exact test.

**Figure 10:**
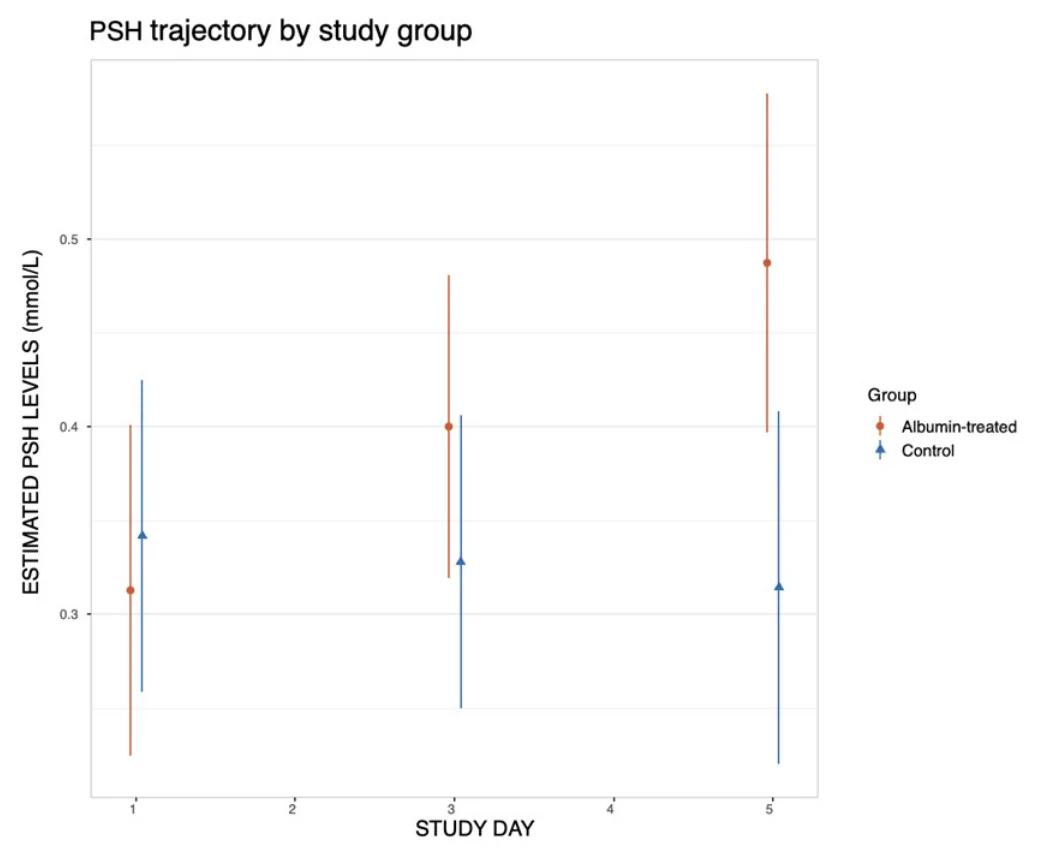
Model-estimated plasma protein sulfhydryl group (PSH) levels in the albumin and control groups (Days 1, 3, and 5). PSH levels increased over time in the albumin group, while declining in the control group. Points represent fitted values from the linear mixed-effects model, and error bars indicate 95% confidence intervals. Group × time interaction from the linear mixed-effects model: *p* < 0.01.

**Figure 11:**
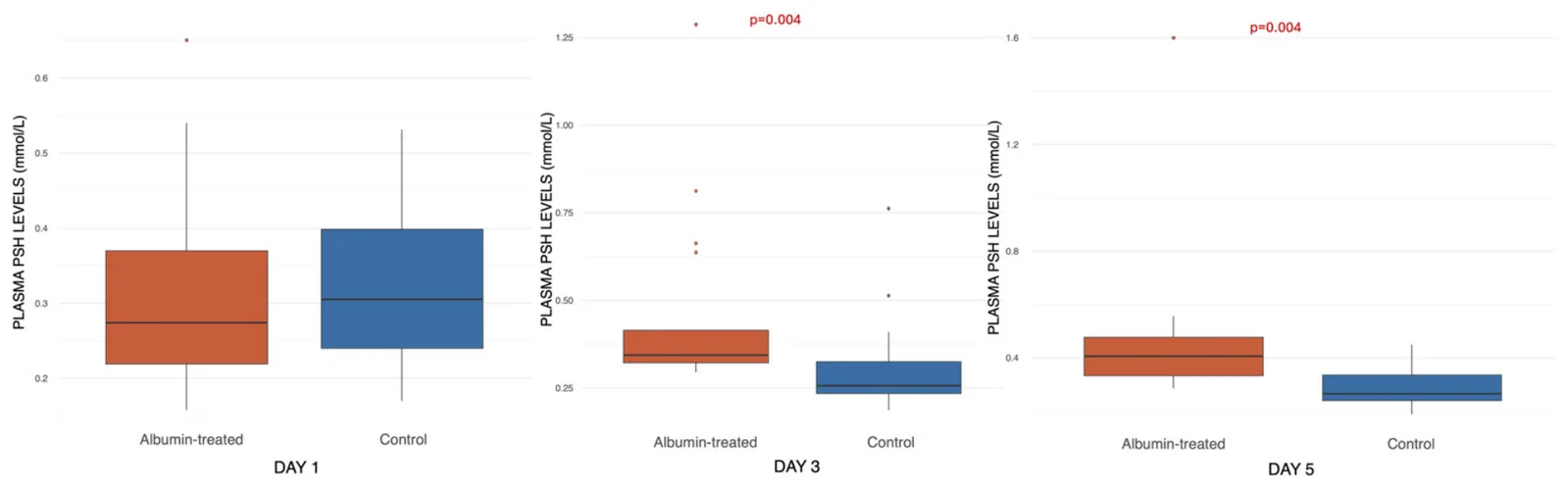
Changes in plasma protein sulfhydryl group (PSH) levels from baseline to Day 5 in the albumin-treated and control groups. Data are presented as boxplots showing the median, interquartile range, and range, with outliers displayed as individual points. PSH levels increased significantly over time in the albumin group, demonstrating a significant difference between the two groups on Days 3 and 5 (Day 1 *p* = 0.492, Day 3 *p* = 0.004, Day 5 *p* = 0.004). Between-group comparisons at each time point were performed using the Wilcoxon rank-sum exact test.

**Figure 12:**
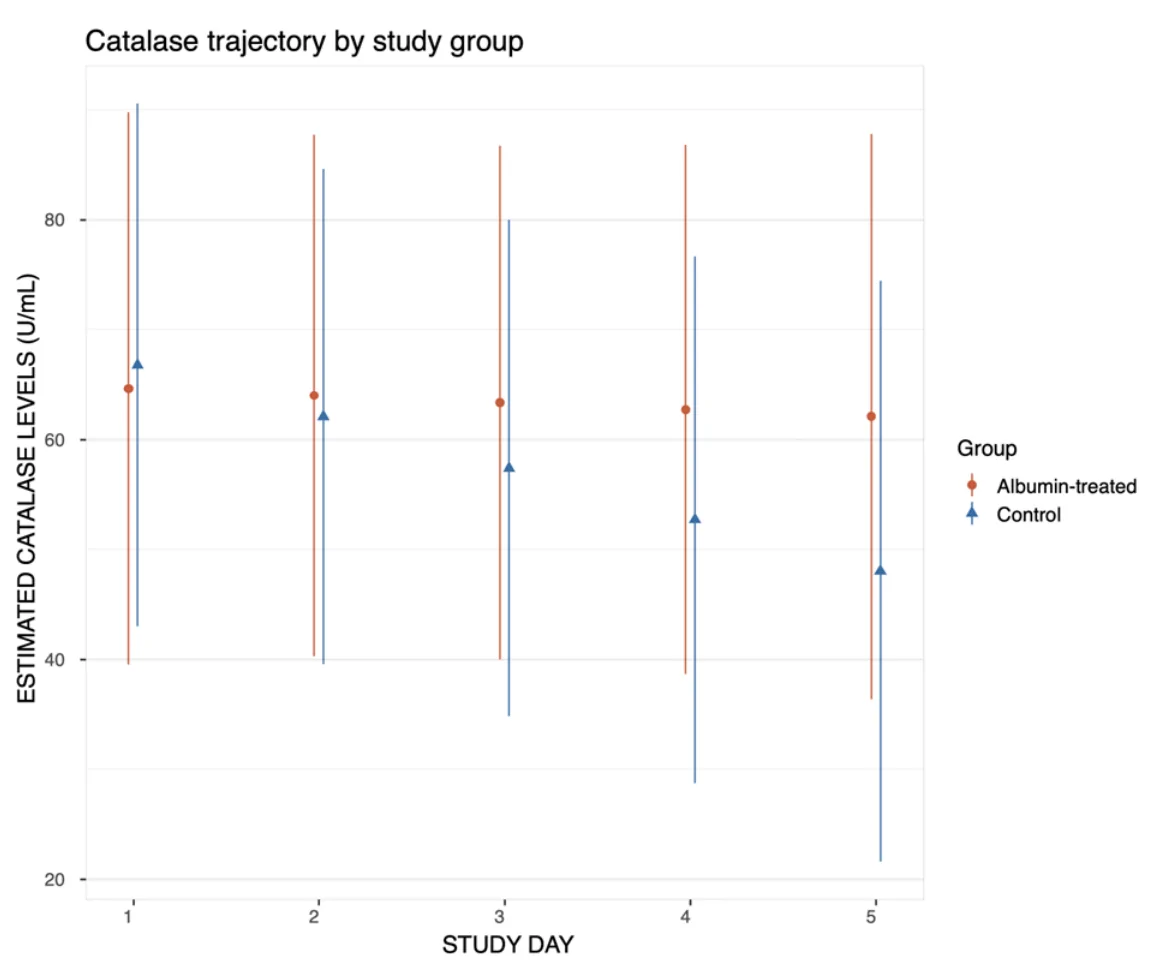
Model-estimated plasma catalase (CAT) activity over the 5-day study period between control and albumin-treated groups. Points represent fitted values from the linear mixed-effects model, and error bars indicate 95% confidence intervals. No significant time–group interaction was observed (*p* = 0.285).

**Figure 13:**
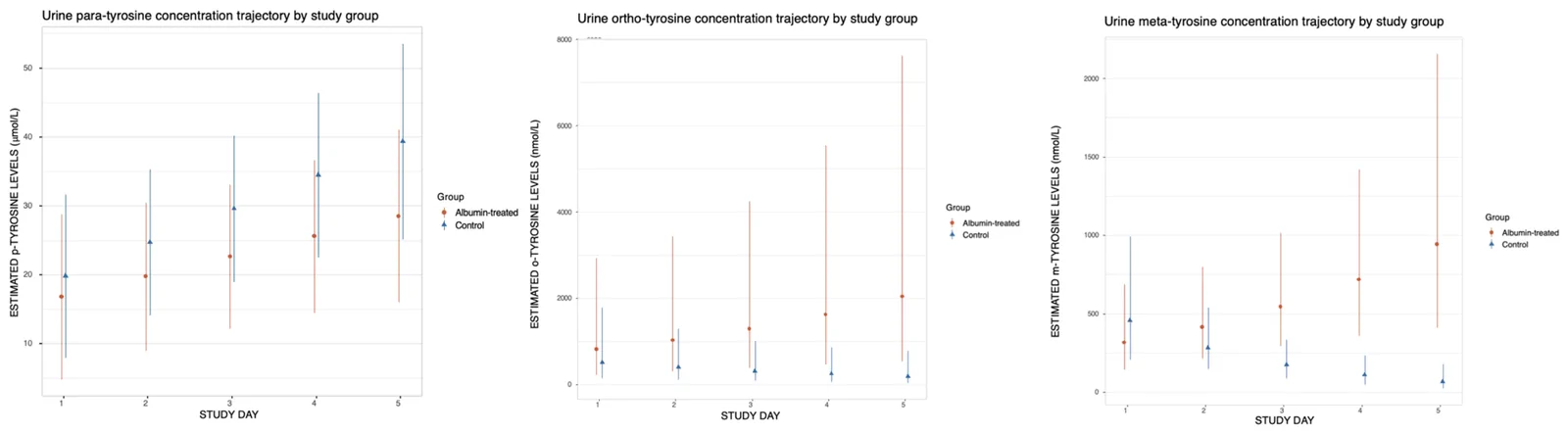
Model-estimated urinary ortho-, meta-, and para-tyrosine (oTyr, mTyr, and pTyr) levels over the 5-day study period in the albumin and control groups. oTyr and mTyr levels increased over time in the albumin group, while declining in the control group. Urinary pTyr levels showed a significant main effect of time (*p* = 0.002), but no significant time–group interaction was observed. Points represent fitted values from the linear mixed-effects model, and error bars indicate 95% confidence intervals. Group × time interaction from the linear mixed-effects model: urinary oTyr *p* = 0.004, urinary mTyr *p* = 0.001.

## Data Availability

All data created or analyzed during this study are available from the corresponding author upon request.

## Supplementary Materials

The following supporting information can be downloaded at https://www.mdpi.com/article/10.3390/biomedicines14102156/s1: Supplementary Table S1: Baseline characteristics; Supplementary Table S2: Linear mixed-effects model results for repeated-measures outcomes; Supplementary Table S3: Linear mixed-effects model results for repeated-measures outcomes; Supplementary Table S4: Logistic regression analysis of 5-day and 30-day mortality; Supplementary Table S4.1: 5-day mortality: Binary logistic regression model; Supplementary Table S4.2: 30-day mortality: Binary logistic regression model; Supplementary Figure S1: Serum albumin concentrations on study Days 3, 4, and 5; Supplementary Figure S2: Comparison of C-reactive protein levels between the albumin and control groups on Days 1, 3, and 5; Supplementary Figure S3: Neutrophil-to-lymphocyte ratio by study group; Supplementary Figure S4: Upper fraction of LAR by study group; Supplementary Figure S5: Lower fraction of LAR by study group.

## Abbreviations

The following abbreviations are used in this manuscript:

CAT	Catalase activity
CI	Confidence interval
CRP	C-reactive protein
ELISA	Enzyme-Linked Immunosorbent Assay
GSH	Glutathione
GSSG	Oxidized glutathione (glutathione disulfide)
ICU	Intensive care unit
IL-1β	Interleukin-1β
IL-6	Interleukin-6
LAR	Leukocyte antisedimentation rate
LAR upper	Upper fraction of leukocyte antisedimentation rate
LAR lower	Lower fraction of leukocyte antisedimentation rate
MAP	Mean arterial pressure
MPO	Myeloperoxidase
mTyr	Meta-tyrosine
NADPH	Nicotinamide adenine dinucleotide phosphate (reduced form)
NLR	Neutrophil-to-lymphocyte ratio
oTyr	Ortho-tyrosine
PCT	Procalcitonin
Phe	Phenylalanine
PSH	Plasma protein sulfhydryl groups
pTyr	Para-tyrosine
ROS	Reactive oxygen species
RNS	Reactive nitrogen species
RP-HPLC	Reversed-Phase High-Performance Liquid Chromatography
SD	Standard deviation
SOFA	Sequential Organ Failure Assessment
SSC	Surviving Sepsis Campaign
TAC	Total antioxidant capacity
TNF-α	Tumor necrosis factor-alpha
