# Supplementary material for: Albumin Supplementation Preserves Systemic Antioxidant Defenses in Critically Ill, Septic Patients: A Prospective Comparative Study
Source: Biomedicines. 2026 Sep 24;14(10):2156. doi: 10.3390/biomedicines14102156 (PMC13701620; doi:10.3390/biomedicines14102156)
Supplement: Supplementary file 1 [file biomedicines-14-02156-s001.zip › biomedicines-4506616-supplementary.pdf]

Supplementary materials

Supplementary Table S1. Baseline characteristics

Variable	***Albumin*** N = 18[1]	***Control*** N = 20[1]	**p-value**
**Age**	64.72 (13.16)	73.75 (13.10)	**0.026[2]**
**Sex[1]**			0.100[3]
Male	12 (67%)	8 (40%)	
Female	6 (33%)	12 (60%)	
**ICU length of stay**	18.06 (18.42)	12.75 (19.49)	0.271[2]
**Mechanical ventilation requirement[1]**	16 (89%)	14 (70%)	0.238[4]
**5-day mortality[1]**	17 (94%)	17 (85%)	0.606[4]
**30-day mortality[1]**	12 (67%)	16 (80%)	0.468[4]
**Renal replacement therapy[1]**			0.535[4]
CRRT alone	4 (22%)	3 (15%)	
CRRT + IHD	1 (5.6%)	0 (0%)	
**Temperature (°C)**	36.91 (0.91)	36.75 (0.56)	0.666[2]
**Noradrenalin dose (mg/24h)**	165.88 (151.74)	154.25 (90.52)	0.731[5]
**Systolic BP (Hgmm)**	121.29 (15.75)	112.79 (12.07)	0.128[2]
**Diastolic BP (Hgmm)**	62.24 (11.11)	54.26 (6.96)	**0.029[2]**
**MAP (Hgmm)**	82.24 (10.62)	74.61 (7.21)	**0.037[2]**
**Heart rate**	80.94 (12.35)	85.41 (14.43)	0.352[2]

**pH**	7.39 (0.07)	7.40 (0.09)	0.739[2]
**Horowitz quotient (paO2/FiO2)**	206.24 (88.14)	259.37 (127.64)	0.154[2]
**Bicarbonate (mmol/L)**	23.22 (6.64)	25.71 (6.45)	0.383[2]
**Sodium (mmol/L)**	137.44 (6.20)	138.75 (5.00)	0.528[2]
**Potassium (mmol/L)**	4.09 (0.54)	4.09 (0.65)	0.907[2]
**Carbamide (mmol/L)**	16.23 (10.85)	14.12 (8.20)	0.737[2]
**Creatinine (µmol/L)**	205.28 (168.75)	161.35 (127.28)	0.640[2]
**Total protein (g/L)**	46.05 (8.48)	45.44 (8.72)	0.236[2]
**Albumin (g/L)**	23.44 (4.05)	25.91 (3.89)	0.154[2]
**Total bilirubin (µmol/L)**	14.27 (13.20)	18.37 (23.70)	0.924[2]
**C-reactive protein (mg/L)**	232.34 (120.03)	229.53 (128.51)	0.851[5]
**Procalcitonin (ng/mL)**	22.65 (22.24)	31.58 (49.21)	0.245[5]
**Leukocyte ($\times 10^9$/L)**	17.35 (8.43)	16.43 (9.24)	0.696[5]
**Hemoglobin (g/L)**	106.22 (17.06)	110.94 (14.37)	0.235[2]
**Hematocrit (%)**	30.67 (4.91)	31.49 (5.14)	0.531[2]
**Platelet ($\times 10^9$/L)**	226.67 (122.41)	193.42 (93.67)	0.773[2]
**NLR**	26.72 (31.97)	20.63 (13.90)	0.762[5]
**LAR upper fraction**	20.69 (11.81)	17.53 (6.20)	0.549[2]
**LAR lower fraction**	9.55 (5.00)	12.38 (7.56)	0.427[5]
**Total antioxidant capacity (µmol/L)**	515.59 (252.38)	406.82 (170.76)	0.167[5]
**Myeloperoxidase (U/L)**	448.60 (295.21)	424.91 (213.90)	0.715[2]

**GSH (μmol/L)**	72.96 (22.77)	90.37 (34.77)	0.112[5]
**PSH (mmol/L)**	0.31 (0.13)	0.33 (0.11)	0.492[2]
**CAT (U/mL)**	63.57 (44.37)	64.07 (72.62)	0.511[2]
**Serum phenylalanine concentration(μmol/L)**	69.07 (30.98)	68.27 (30.61)	0.897[5]
**Serum para-tyrosine concentration (μmol/L)**	35.01 (16.27)	38.13 (20.65)	0.696[5]
**Serum ortho-tyrosine concentration (nmol/L)**	388.83 (600.34)	283.94 (428.77)	0.965[5]
**Serum meta-tyrosine concentration (nmol/L)**	125.28 (153.92)	54.84 (66.20)	0.067[5]
**Urine phenylalanine concentration (μmol/L)**	125.37 (154.36)	129.31 (103.11)	0.653[5]
**Urine para-tyrosine concentration (μmol/L)**	15.37 (14.83)	19.05 (14.72)	0.539[5]
**Urine ortho-tyrosine concentration (nmol/L)**	4073.92 (6157.76)	5567.33 (15206.07)	0.806[5]
**Urine meta-tyrosine concentration (nmol/L)**	1117.14 (1706.97)	1463.15 (2421.54)	0.999[5]
**IL-1β (pg/mL)**	10.44 (2.88)	13.31 (13.71)	0.965[2]
**IL-6 (pg/mL)**	1,256.51 (1,906.56)	4,482.37 (11,302.58)	0.633[5]

Values are mean (SD)
[1] n (%)
[2] Wilcoxon rank sum test
[3] Pearson's Chi-squared test
[4] Fisher's exact test
[5] Wilcoxon rank sum exact test

Supplementary Table S2. Linear mixed-effects model results for repeated-measures outcomes.

Outcome	Observations, *n*	Age effect, β (SD)	Age effect, *p*	Day effect, β (SD)	Day effect, *p*	Group effect (control vs. albumin), β (SD)	Group effect, *p*	Day × Group interaction, β (SD)	Day × Group interaction, *p*
**Albumin**	143	-0.014 (3.186)	0.763	2.778 (0.283)	**<0.001**	3.903 (1.679)	**0.022**	-3.096 (0.420)	**<0.001**
**Total protein**	130	-0.076 (0.089)	0.405	1.721 (0.316)	**<0.001**	3.104 (2.726)	0.260	-2.373 (0.456)	**<0.001**
**CRP**	159	1.883 (1.216)	0.1300	-31.711 (6.713)	**<0.001**	-13.445 (41.881)	0.749	9.667 (9.762)	0.324
**Procalcitonin**	159	-0.228 (0.362)	0.533	-4.133 (1.006)	**<0.001**	16.283 (10.582)	0.130	-2.483 (1.478)	0.095
**MPO**	112	-4.285 (2.729)	0.125	8.888 (14.340)	0.537	20.053 (91.080)	0.826	3.916 (21.345)	0.854
**IL-1**	161	-0.107 (0.099)	0.288	-0.034 (0.613)	0.955	0.831 (3.630)	0.819	0.981 (0.898)	0.276
**IL-6**	98	51.64 (44.65)	0.254	-278.83 (454.69)	0.542	3260.23 (2154.61)	0.134	-915.53 (661.70)	0.171

**TAC**	98	3.549 (2.137)	0.105	-26.921 (10.374)	**0.011**	-132.255 (70.312)	0.064	-11.943 (15.405)	0.441
**GSH**	98	-0.687 (0.303)	**<0.05**	10.711 (2.350)	**<0.001**	30.189 (12.279)	**<0.05**	-14.155 (3.454)	**<0.001**
**PSH**	98	-0.003 (0.002)	0.082	0.043 (0.002)	0.082	0.079 (0.069)	0.253	-0.050 (0.014)	**<0.01**
**Catalase**	101	-0.441 (0.626)	0.486	-0.638 (2.523)	0.801	6.173 (19.497)	0.753	-4.048 (3.754)	0.285
**Serum phenylalanine**	162	-0.695 (0.249)	**0.008**	-1.501 (1.609)	0.352	6.133 (9.275)	0.509	-0.878 (2.329)	0.706
**Serum p-tyrosine**	162	-0.494 (0.229)	**0.037**	2.044 (1.056)	0.055	6.204 (7.476)	0.409	-1.339 (1.533)	0.383
**Serum m-tyrosine**	162	0.231 (1.191)	0.847	-11.980 (4.926)	**0.016**	-70.105 (37.615)	0.066	6.671 (7.155)	0.352
**Serum o-tyrosine**	162	7.237 (10.516)	0.495	138.591 (52.965)	**0.009**	-54.216 (352.824)	0.878	-110.721 (76.829)	0.151
**Urine phenylalanine**	125	-4.119 (2.430)	0.099	6.861 (10.719)	0.523	-1.752 (75.051)	0.981	-12.110 (16.616)	0.467
**Urine p-tyrosine**	125	-0.441 (0.305)	0.157	2.925 (1.619)	0.074	1.050 (10.239)	0.916	1.956 (2.510)	0.437
**Urine m-tyrosine**	125	1.858 (20.791)	0.929	350.280 (132.755)	**0.009**	1025.135 (738.840)	0.168	-677.286 (204.619)	**0.001**

**Urine o-tyrosine**	125	58.97 (158.95)	0.712	4000.08 (959.54)	**<0.001**	6085.85 (5501.81)	0.271	-4379.86 (1481.70)	**0.003**
**NLR**	162	-0.211 (0.180)	0.250	-1.706 (0.894)	0.058	-0.588 (6.014)	0.922	-0.281 (1.311)	0.830
**LAR upper part**	118	-0.052 (0.094)	0.580	-1.091 (0.630)	0.086	-2.171 (3.548)	0.542	0.570 (0.879)	0.518
**LAR lower part**	117	0.037 (0.073)	0.616	-0.001 (0.420)	0.999	1.668 (2.550)	0.515	-0.422 (0.568)	0.460
**Carbamide**	162	0.258 (0.085)	**0.005**	-0.697 (0.352)	**0.049**	-4.455 (2.678)	0.101	0.056 (0.501)	0.911
**Creatinine**	162	1.811 (1.263)	0.160	-27.852 (4.891)	**<0.001**	-81.580 (39.014)	**0.041**	10.977 (6.956)	0.117
**Noradrenaline dose**	123	0.389 (1.118)	0.730	-31.304 (8.831)	**<0.001**	-6.506 (40.110)	0.871	7.827 (11.145)	0.484

Abbreviations: CRP, C-reactive protein; MPO, myeloperoxidase; IL, interleukin; TAC, total antioxidant capacity; GSH, glutathione; PSH, protein sulfhydryl groups; NLR, neutrophil-to-lymphocyte ratio; LAR, leukocyte antisedimentation rate.

Notes: Results are from linear mixed-effects models fitted by restricted maximum likelihood, with patient as a random intercept. *p*-values for age, day, group, and day × group interaction were obtained from Type III analysis of variance with Satterthwaite's approximation. β coefficients are shown from the corresponding fixed-effects models. The albumin group was used as the reference category; therefore, the day effect (β) represents the slope in the albumin group, and the day × group interaction term represents the difference in slope for the control group relative to the albumin group.

Supplementary Table S3. Linear mixed-effects model results for repeated-measures outcomes.

Outcome	Age effect, β (SE)	Age effect, *p*	Day effect, β (SE)	Day effect, *p*	Group effect (control vs. albumin), β (SE)	Group effect, *p*	Day × Group interaction, β (SE)	Day × Group interaction, *p*
**pTyr 24-h urinary excretion**	−0.723 (0.735)	0.333	1.418 (3.884)	0.716	13.380 (23.696)	0.574	**14.599 (5.763)**	**0.013**
**mTyr 24-h urinary excretion**	29.376 (60.321)	0.629	−244.947 (334.037)	0.465	40.266 (1976.286)	0.984	−610.225 (495.275)	0.221
**oTyr 24-h urinary excretion**	181.496 (541.506)	0.739	**8413.526 (3299.290)**	**0.012**	8554.288 (18414.745)	0.644	**−9901.315 (4884.353)**	**0.046**
**Serum pTyr/Phe**	−0.002 (0.005)	0.601	0.019 (0.027)	0.482	−0.096 (0.166)	0.564	0.016 (0.040)	0.680
**Serum mTyr/Phe**	0.016 (0.027)	0.557	**−0.390 (0.170)**	**0.024**	**−2.135 (1.003)**	**0.036**	0.283 (0.246)	0.253
**Serum oTyr/Phe**	0.213 (0.209)	0.314	1.944 (1.275)	0.130	−5.006 (7.574)	0.510	−0.250 (1.847)	0.893
**Urinary pTyr/Phe**	−0.016 (0.010)	0.105	0.008 (0.071)	0.911	−0.157 (0.371)	0.674	**0.236 (0.109)**	**0.033**
**Urinary mTyr/Phe**	0.488 (0.827)	0.559	3.333 (4.031)	0.410	11.531 (26.284)	0.662	−11.190 (6.284)	0.078

**Urinary oTyr/Phe**	1.750 (4.943)	0.725	38.871 (24.408)	0.115	−4.830 (157.788)	0.976	−45.767 (38.038)	0.232
**Serum oTyr/pTyr**	0.283 (0.266)	0.294	1.680 (1.354)	0.217	−4.584 (8.965)	0.610	−1.388 (1.964)	0.481
**Serum mTyr/pTyr**	0.026 (0.035)	0.456	**−0.517 (0.219)**	**0.020**	−2.047 (1.276)	0.112	0.123 (0.317)	0.699
**Urinary oTyr/pTyr**	3.668 (15.975)	0.820	**256.490 (94.531)**	**0.008**	301.348 (548.057)	0.584	−266.789 (146.065)	0.071
**Urinary mTyr/pTyr**	0.024 (1.789)	0.989	6.764 (8.251)	0.414	15.884 (55.978)	0.778	**−27.024 (12.830)**	**0.038**

Abbreviations: β, estimated fixed-effect coefficient; SE, standard error; pTyr, para-tyrosine; mTyr, meta-tyrosine; oTyr, ortho-tyrosine; Phe, phenylalanine; LMM, linear mixed-effects model.

Notes: Values are presented as estimated fixed-effect coefficients, β (SE). Linear mixed-effects models included age, study day, treatment group, and the Day × Group interaction as fixed effects, with a random intercept for patient ID to account for repeated measurements. The group coefficient represents the estimated difference between the control and albumin groups according to the reference-group coding used in the model. The Day × Group interaction indicates whether the change in the outcome over time differed between the groups. Two-sided p values < 0.05 were considered statistically significant.

Supplementary Table S4. Logistic regression analysis of 5-day and 30-day mortality.

Outcome	Albumin group, *n/N*(%)	Control group, *n/N*(%)	Adjusted OR for control vs. albumin (95% CI)	*p*-Value
**5-day mortality**	1/18 (5.6)	3/20 (15.0)	3.09 (0.24–40.08)	0.388
**30-day mortality**	6/18 (33.3)	4/20 (20.0)	0.57 (0.11–2.83)	0.490

Abbreviations: CI, confidence interval; OR, odds ratio.

Notes: Odds ratios were obtained from multivariable logistic regression models adjusted for age and sex. For the 5-day mortality model, the 95% CI shown was derived from the available generalized linear model coefficients using the Wald method. Neither age nor sex was significantly associated with 5-day mortality (p = 0.974 and p = 0.885, respectively) or 30-day mortality (p = 0.810 and p = 0.750, respectively).

Supplementary Table S4.1: 5-day mortality: Binary logistic regression model

Variable	N	Cases	OR	95% CI	p-value	VIF
**Age**	38	4	1.00	0.925, 1.10	0.974	1.11
**Sex**	38	4				1.07
male	20	2	1.00	—		
female	18	2	0.853	0.085, 8.33	0.885	
**Group**	38	4				1.18
albumin	18	1	1.00	—		
control	20	3	3.09	0.287, 76.1	0.388	

Abbreviations: CI = Confidence Interval, OR = Odds Ratio, VIF = Variance Inflation Factor

Null deviance = 25.6; Null df = 37; Log-likelihood = -12.3; AIC = 32.6; BIC = 39.2; Deviance = 24.6; Residual df = 34; No. Obs. = 38

Supplementary Table S4.2: 30-day mortality: Binary logistic regression model

Variable	N	Cases	OR	95% CI	p-value	VIF
**Age**	38	10	0.993	0.938, 1.05	0.806	1.12
**Sex**	38	10				1.07
male	20	6	1.00	—		
female	18	4	0.783	0.160, 3.66	0.754	
**Group**	38	10				1.19
albumin	18	6	1.00	—		
control	20	4	0.568	0.106, 2.83	0.490	

Abbreviations: CI = Confidence Interval, OR = Odds Ratio, VIF = Variance Inflation Factor

Null deviance = 43.8; Null df = 37; Log-likelihood = -21.4; AIC = 50.8; BIC = 57.3; Deviance = 42.8; Residual df = 34; No. Obs. = 38

Supplementary figures

Supplementary Figure S1

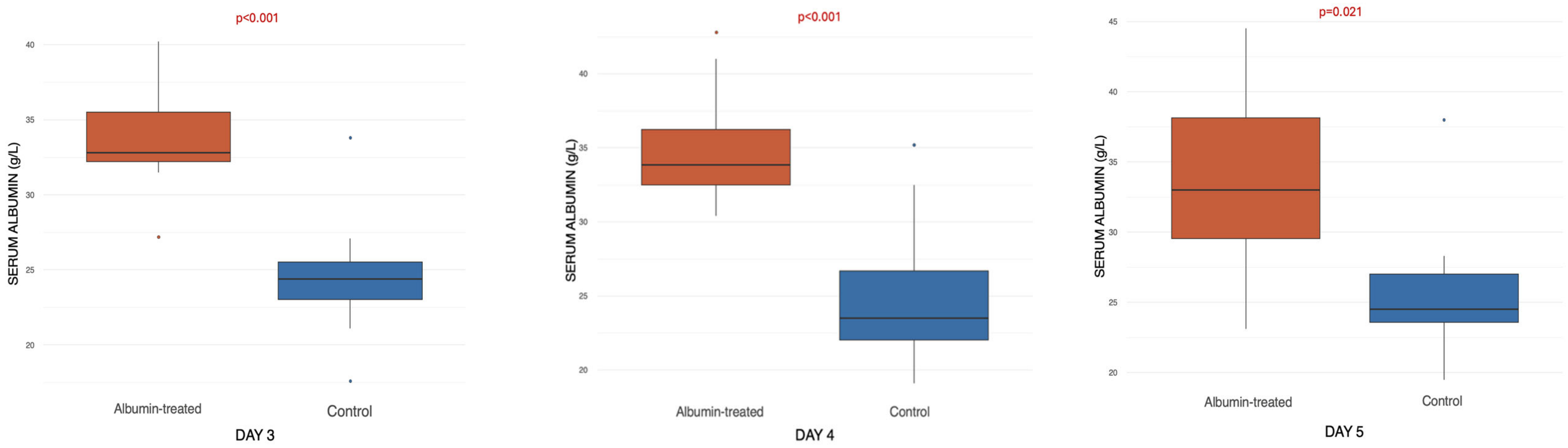

Figure S1. Serum albumin concentrations on study day 3, 4 and 5. Boxplots show the median, interquartile range, whiskers, and outliers. A statistically significant separation in plasma albumin levels emerged between Day 3 (p<0.001) and Day 5 (p=0.021). Between-group comparisons at each time point were performed using the Wilcoxon rank-sum exact test.

Supplementary Figure S2

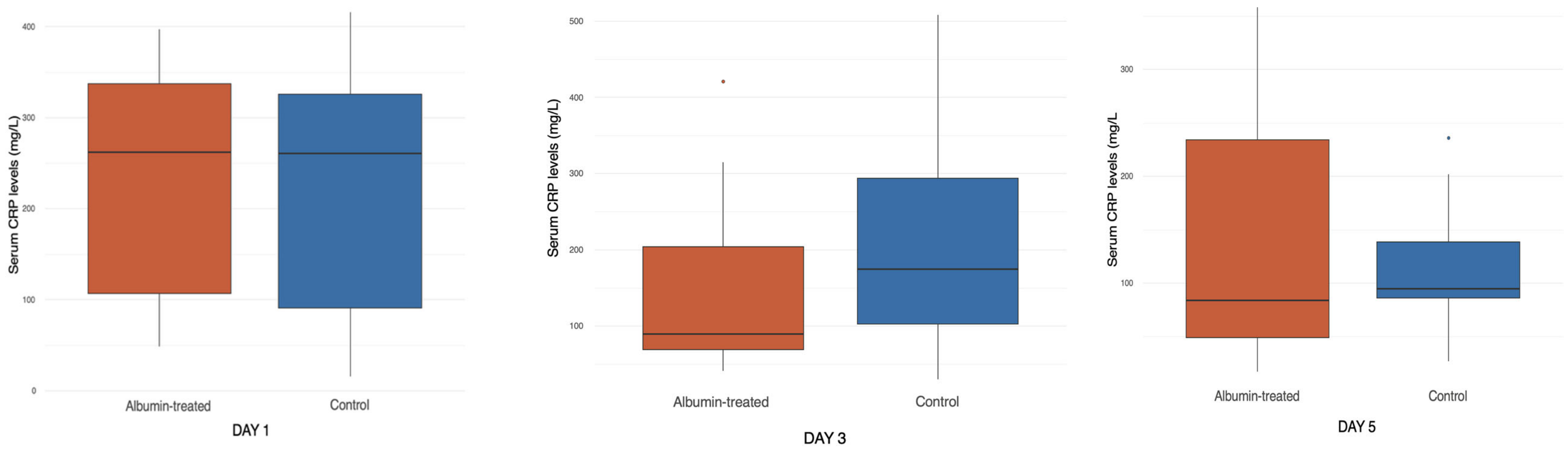

Figure S2. Comparison of C-reactive protein levels between the albumin and control groups pn Day 1, 3 and 5. Data are presented as boxplots showing the median, interquartile range, and range with outliers displayed as individual points. CRP levels demonstrated no significant difference between the two groups at any time point of the study period (Day 1 P-0.851, Day 3 p=0.184, Day 5 p=0.820). Between-group comparisons at each time point were performed using the Wilcoxon rank-sum exact test.

Supplementary Figure S3

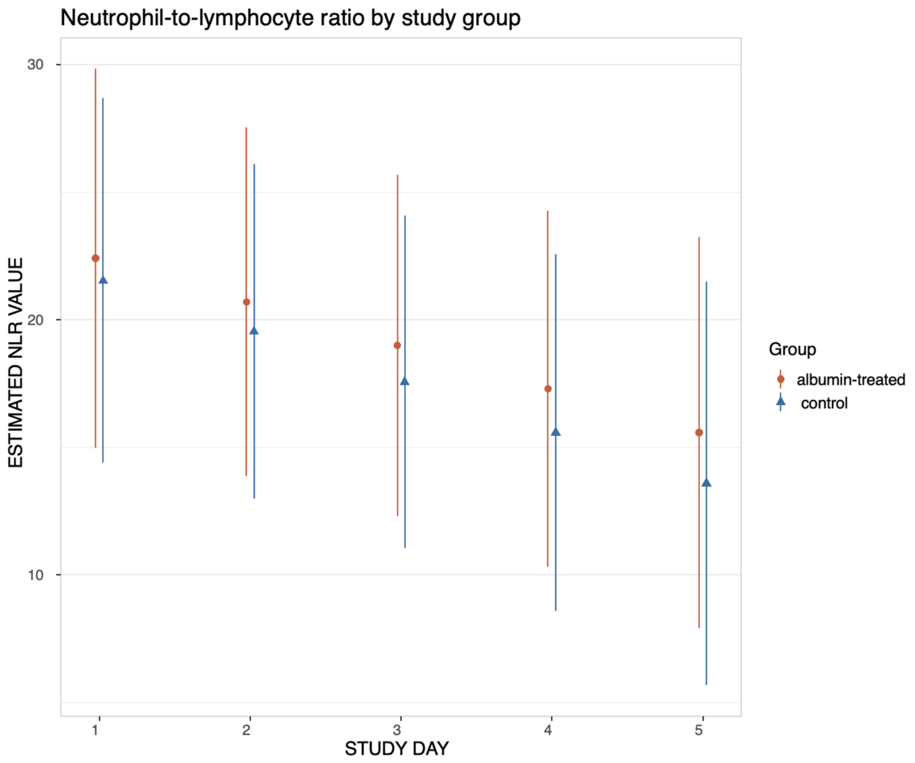

Figure S3. Effect of 5-day albumin administration on neutrophil-to-lymphocyte ratio (NLR) in control and albumin-treated groups (Days 1–5). Points represent fitted values from the linear mixed-effects model, and error bars indicate 95% confidence intervals. A significant main effect of time was observed (p=0.005), whereas the time x group interaction was not significant (p=0.830).

Supplementary Figure S4

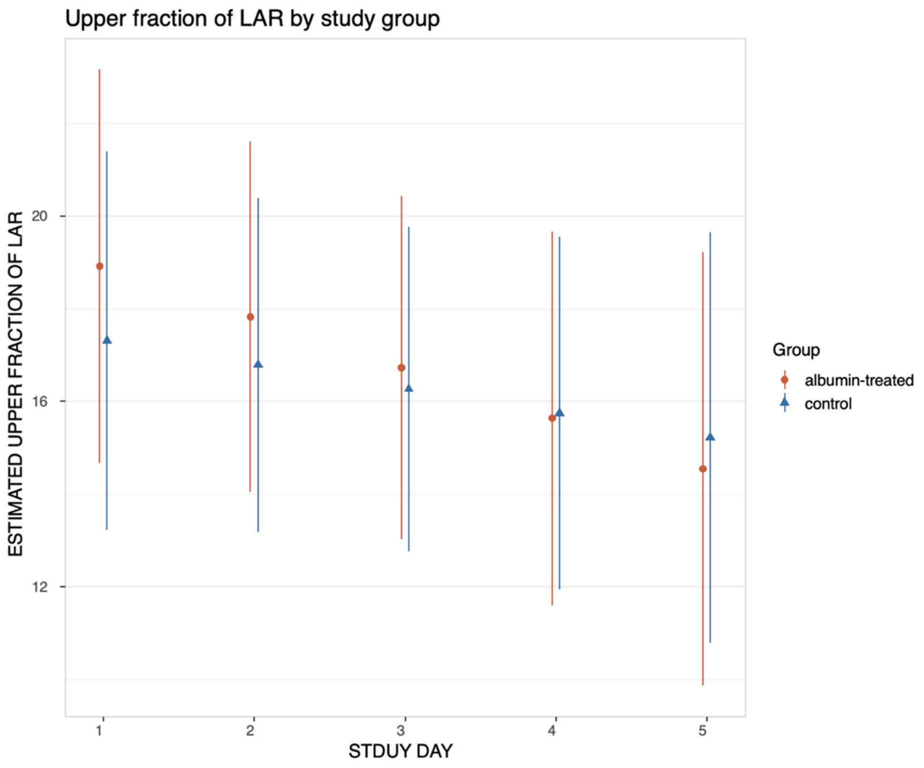

Figure S4. Effect of 5-day albumin administration on the change of upper fraction of leukocyte antisedimentation rate (LAR) in control and albumin-treated groups (Days 1–5). Points represent fitted values from the linear mixed-effects model, and error bars indicate 95% confidence intervals. No significant main effect of time or time-group interaction was observed.

Supplementary Figure S5

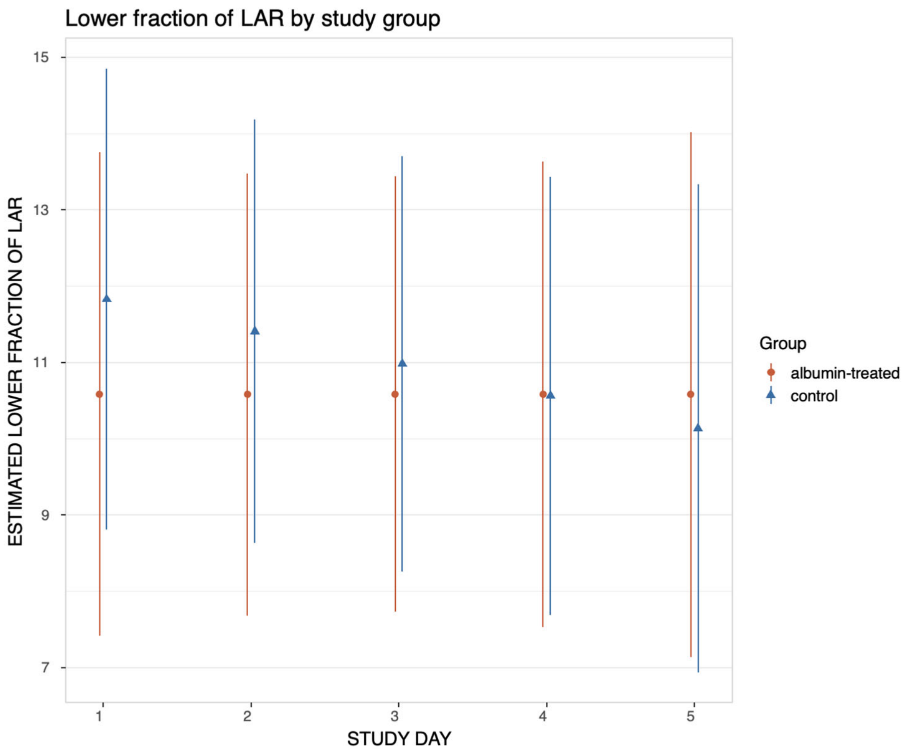

Figure S5. Effect of 5-day albumin administration on the change of lower fraction of leukocyte antisedimentation rate (LAR) in control and albumin-treated groups (Days 1–5). Points represent fitted values from the linear mixed-effects model, and error bars indicate 95% confidence intervals. No significant main effect of time or time-group interaction was observed.
